# Neurotoxic β-amyloid oligomers cause mitochondrial dysfunction—the trigger for PANoptosis in neurons

**DOI:** 10.3389/fnagi.2024.1400544

**Published:** 2024-05-14

**Authors:** Xiangyuan Meng, Qi Song, Zinan Liu, Xinpeng Liu, Yujie Wang, Jinyu Liu

**Affiliations:** Department of Toxicology, School of Public Health, Jilin University, Changchun, China

**Keywords:** Alzheimer's disease, Aβ oligomer, mitochondria, PANoptosis, neuron

## Abstract

As the global population ages, the incidence of elderly patients with dementia, represented by Alzheimer's disease (AD), will continue to increase. Previous studies have suggested that β-amyloid protein (Aβ) deposition is a key factor leading to AD. However, the clinical efficacy of treating AD with anti-Aβ protein antibodies is not satisfactory, suggesting that Aβ amyloidosis may be a pathological change rather than a key factor leading to AD. Identification of the causes of AD and development of corresponding prevention and treatment strategies is an important goal of current research. Following the discovery of soluble oligomeric forms of Aβ (AβO) in 1998, scientists began to focus on the neurotoxicity of AβOs. As an endogenous neurotoxin, the active growth of AβOs can lead to neuronal death, which is believed to occur before plaque formation, suggesting that AβOs are the key factors leading to AD. PANoptosis, a newly proposed concept of cell death that includes known modes of pyroptosis, apoptosis, and necroptosis, is a form of cell death regulated by the PANoptosome complex. Neuronal survival depends on proper mitochondrial function. Under conditions of AβO interference, mitochondrial dysfunction occurs, releasing lethal contents as potential upstream effectors of the PANoptosome. Considering the critical role of neurons in cognitive function and the development of AD as well as the regulatory role of mitochondrial function in neuronal survival, investigation of the potential mechanisms leading to neuronal PANoptosis is crucial. This review describes the disruption of neuronal mitochondrial function by AβOs and elucidates how AβOs may activate neuronal PANoptosis by causing mitochondrial dysfunction during the development of AD, providing guidance for the development of targeted neuronal treatment strategies.

## 1 Introduction

According to the “2022 World Alzheimer's Disease Report,” more than 550 million people worldwide suffer from Alzheimer's disease (AD) or related conditions (Serge Gauthier et al., 2022). It is estimated that by 2030 this number will increase to 820 million and that by 2050 it will increase to 1.38 billion. AD is an incurable central nervous system disorder that worsens over time, impairing thinking and memory. Ultimately, AD patients lose the ability to care for themselves, imposing a significant economic burden on families and society. AD is the most common cause of dementia in elderly people, accounting for ~60–70% of cases (https://www.who.int/news-room/fact-sheets/detail/dementia). Clinical manifestations of AD include declining memory, unclear speech, confusion, impairment of judgment, and other abnormalities in brain function, accompanied by behavioral and psychological changes (Tarawneh and Holtzman, 2012).

The pathological characteristics of AD at the histological level include changes in brain tissue structure and function that are caused by degenerative changes in neurons (Nelson et al., 2012). At the molecular level, one of the most characteristic pathologies of AD is β-amyloidosis, a condition in which β-amyloid protein (Aβ) accumulates in various areas of brain tissue. There, Aβ further aggregates to form Aβ plaques, leading to degenerative changes and even death of neurons and ultimately resulting in dysfunction of the nervous system (Guo et al., 2020). The abnormal death of neurons is a major cause of AD. However, research on neuronal death in AD has rarely been comprehensively reexamined since 2000 (Serrano-Pozo et al., 2017). Convincing evidence suggests that the aggregation and accumulation of Aβ are decisive events leading to AD (Vickers et al., 2016). Soluble Aβ oligomers (AβO) are believed to be more harmful than Aβ fibrils and plaques and are considered the main causes of various toxic effects, such as decreased cognitive ability and neuronal death, in brains affected by AD (Lambert et al., 1998; Cline et al., 2018). Therefore, a thorough investigation of the toxic mechanisms by which AβOs induce neuronal death will not only contribute to the discovery of the pathological basis of AD but also holds promise for identifying new therapeutic targets for AD and creating new breakthroughs in AD treatment strategies.

Neuronal survival depends on the proper functioning of mitochondria (Cheng et al., 2022). Mitochondrial dysfunction is a core feature of neurodegenerative diseases (Swerdlow, 2018; Cheng et al., 2022). Mitochondria are membrane-bound organelles that are found in the cytoplasm of eukaryotic cells and produce energy in the form of adenosine triphosphate (ATP) through oxidative phosphorylation. Mitochondria undergo dynamic changes in morphology and function, and changes in their morphology trigger downstream functional alterations. These dynamic changes regulate the macroscopic and microscopic morphology of mitochondria by controlling mitochondrial fission and fusion. At the macroscopic level, mitochondria exhibit a dynamic equilibrium between fission and fusion (Zacharioudakis and Gavathiotis, 2023). This disruption of homeostasis can lead to excessive fragmentation of mitochondria, thereby reducing the integrity of the mitochondrial network within the cell. Changes in mitochondrial morphology directly affect the oxidative phosphorylation (OXPHOS) process within mitochondria, leading to a reduction in mitochondrial quality. Mitochondrial autophagy, also known as mitophagy, works in concert with mitochondrial biogenesis to eliminate depolarized or damaged mitochondria and generate new, healthy mitochondria, thereby maintaining mitochondrial quality and quantity (Guo et al., 2023c). Therefore, coordination among mitochondrial dynamics, oxidative phosphorylation, mitochondrial biogenesis, and mitophagy ensures the proper functioning of mitochondria (Monzel et al., 2023).

Compared to Aβ monomers, AβOs have a greater affinity for biological membrane lipid bilayers. Not only do AβOs directly insert into lipid bilayers, altering neuronal cell membrane structure and causing the formation of membrane pores; they are also internalized into neurons via endocytic vesicles, accelerating their aggregation in the lysosomal acidic environment. Increasing evidence suggests that AβOs impair mitochondrial function by disrupting mitochondrial dynamics and mitochondrial cristae structure, decreasing mitochondrial quantity and quality, causing defects in mitochondrial DNA (mtDNA), and inhibiting the mitochondrial electron transport chain (ETC; Baloyannis, 2006; Krishnan et al., 2012; Mckenna et al., 2016; Arrázola et al., 2017; Sorrentino et al., 2017; Perez Ortiz and Swerdlow, 2019). The resulting mitochondrial dysfunction triggers a series of events that include but are not limited to cell apoptosis, pyroptosis, and necrosis, suggesting that mitochondria are the primary targets of the negative effects of AβO.

The recently proposed process of PANoptosis is a unique programmed cell death (PCD) that is activated by specific triggers. PANoptosis exhibits molecular characteristics of pyroptosis, apoptosis, and/or necroptosis and cannot be solely explained by any single PCD pathway (Wang and Kanneganti, 2021; Zhu et al., 2023). Currently, there is no experimental evidence suggesting that PANoptosis occurs in patients with AD or in AD model animals, and there is also a lack of pioneering *in vitro* research results. Considering that mitochondria are the primary targets of AβOs in neurons, mitochondrial dysfunction is potentially closely associated with the initiation of PANoptosis. Therefore, taking mitochondria as a starting point, elucidating the pathology and the biological basis of AD from the perspective of PANoptosis will aid in clarifying the pathogenesis of AD.

## 2 The production and aggregation of Aβ

**Figure 1:**
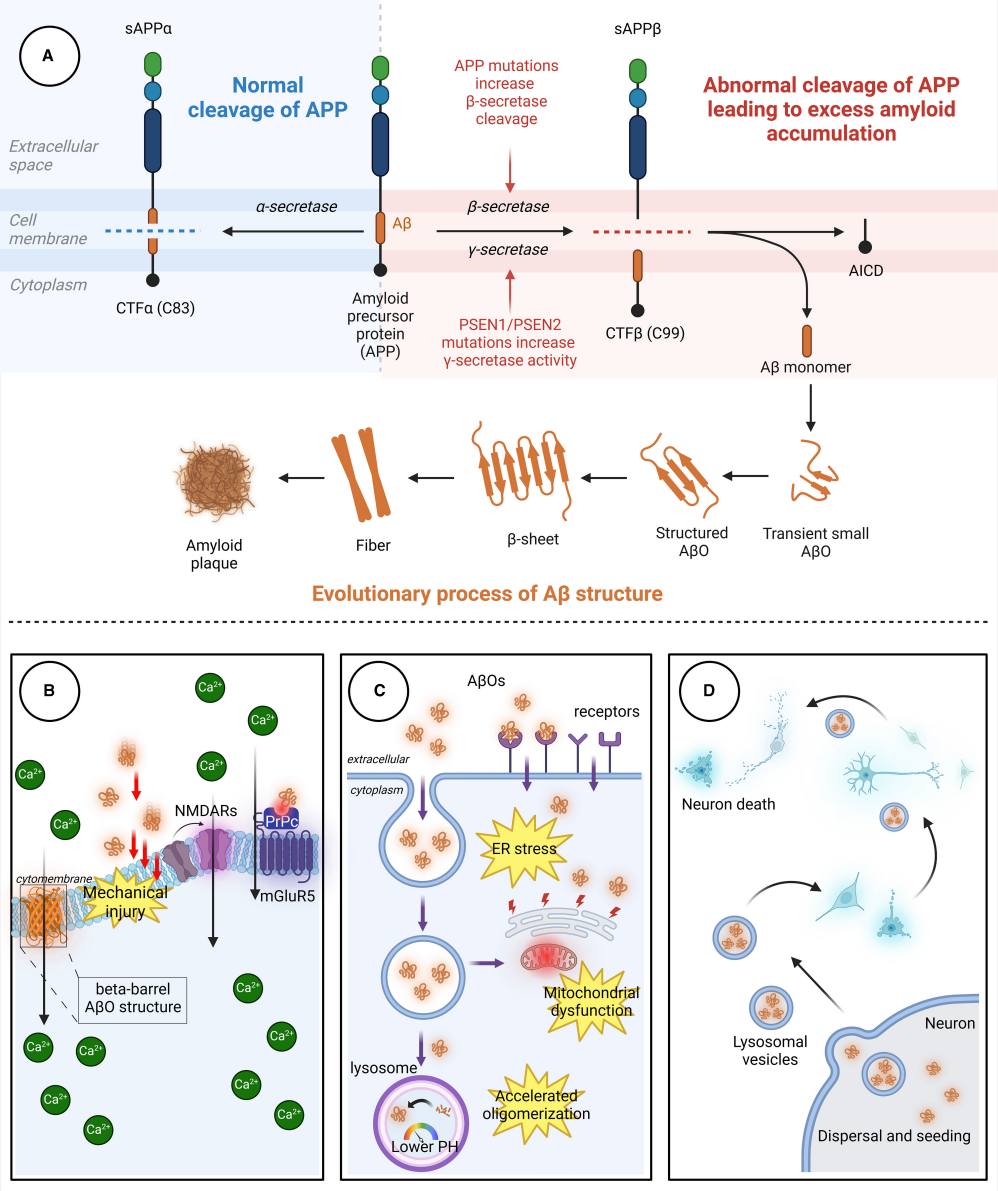
The process through which Aβ aggregates and the pathways through which AβOs cause neuronal damage. **(A)** In the non-amyloidogenic pathway, APP is initially cleaved by α-secretase to generate sAPPα and the carboxyl-terminal fragment CTFα. In the amyloidogenic pathway, APP is sequentially cleaved by β-secretase and γ-secretase. β-Secretase cleavage produces the 99-amino acid CTFβ fragment, which is further cleaved by γ-secretase to release the Aβ peptide. The release of Aβ leads to the spontaneous aggregation of Aβ monomers into soluble AβOs, which then continuously aggregate to form protofibrils with rapidly increasing β-fold structures. Finally, the fibrils aggregate to form Aβ plaques with distinct clinicopathological features. **(B)** AβOs can induce neurotoxicity by directly perforating the membrane or by binding to receptors, leading to intracellular Ca^2+^ influx. **(C)** AβOs can be internalized into neurons in an endocytic manner through specific receptors on neuronal membranes, causing damage to organelles. **(D)** AβOs exhibit a prion-like spreading effect, enabling transmission between neurons. Aβ, β-amyloid protein; AβO, Aβ oligomer; APP, amyloid precursor protein; AICD, APP intracellular domain; CTFα, C-terminal fragment α; CTFβ, C-terminal fragment β; ER, endoplasmic reticulum; mGluR5, metabotropic glutamate receptor 5; NMDAR, N-methyl-D-aspartic acid receptor; PrPc, cellular prion protein; sAPPα, soluble APP fragment α; sAPPβ, soluble APP fragment β.

Aβ, a polypeptide consisting of 39–43 amino acids, is formed through the proteolysis of amyloid precursor protein (APP) by β-secretase and γ-secretase. APP, a transmembrane protein that is highly expressed in the nervous system, undergoes proteolytic cleavage through both amyloidogenic and non-amyloidogenic pathways (Figure 1). In the amyloidogenic pathway, β-secretase and γ-secretase sequentially cleave APP to generate Aβ peptides, predominantly Aβ_40_ and Aβ_42_. β-Site amyloid precursor protein cleaving enzyme-1 (BACE1), a key enzyme, initiates this process, and γ-secretase completes it. The acidic environment of endosomes and lysosomes is crucial for β-secretase activity. In the non-amyloidogenic pathway, α-secretase cleaves APP, producing non-toxic fragments that inhibit Aβ formation. Additionally, soluble APP fragment α (sAPPα) generated in this pathway has neuroprotective effects and opposes BACE1 activity, impacting cognitive functions and neuronal survival.

Aβ is an amphipathic peptide with a strongly hydrophilic N-terminus and a hydrophobic C-terminus (Han and He, 2018). Due to the presence of hydrophobic amino acids, the release of Aβ fragments initially leads to the spontaneous aggregation of Aβ monomers into soluble Aβ oligomers, followed by aggregation of the oligomers to form insoluble protofibrils (Hayden and Teplow, 2013). The protofibrils subsequently aggregate to form Aβ plaques. Aβ monomers aggregate through a mechanism called “nucleation-dependent polymerization,” which includes three key stages: nucleation, elongation, and maturation (Chiti and Dobson, 2017; Okumura and Itoh, 2022). In the nucleation stage, conformational changes occur in Aβ monomers, exposing a hydrophobic Lys16-Phe20 sequence in the nucleation core region (Walsh et al., 1999). Then, two Aβ monomers self-recognize through specific binding sites to form dimers with an antiparallel β-fold structure. These dimers undergo conformational changes and gradually aggregate into “nucleus”-like metastable oligomers, marking the beginning of the nucleation stage. In the elongation stage, numerous Aβ oligomers enter a state of accelerated growth, continuously aggregating to form protofibrils with rapidly increasing β-fold structures (Ono and Watanabe-Nakayama, 2021). Finally, in the maturation period, protofibrils aggregate to form β-amyloid protein plaques.

## 3 The neurotoxicity of AβOs

The appearance of Aβ plaques in the brain, known as senile plaques, is a major hallmark of AD (Walker, 2020). Despite the fact that Aβ plaques are considered pathological markers of AD, soluble AβOs, especially oligomers formed by 2–50 monomers, are believed to be the main causes of the various neurotoxic effects that lead to cognitive decline in the AD brain. Recently, a 20-year multicenter nested case-control study revealed that as cognitive function declines, the rate of change in Aβ_42_ and Aβ_42_/Aβ_40_ in the cerebrospinal fluid of AD patients initially accelerates significantly (Jia et al., 2024). However, with time, the rate of change slows, suggesting that in the late stages of AD, Aβ aggregates into large protofibrils and that its toxic mechanisms are complete. The accumulating evidence to date supports the following statements: (1) monomeric Aβ has important biological functions at physiological concentrations (Kumar et al., 2016; Brothers et al., 2018); (2) in the elderly population, there is a large accumulation of Aβ plaques in the brain in the absence of severe cognitive impairment, while AD patients, despite having a small amount of Aβ aggregation, exhibit more severe dementia symptoms (Tomiyama et al., 2008; Shimada et al., 2011); and (3) the plasma AβO concentration shows good accuracy as a marker in screening for AD (Lue et al., 1999; Mclean et al., 1999; Näslund et al., 2000; Steinerman et al., 2008; Lesné et al., 2013).

The APP gene is ancient and highly conserved; the Aβ sequences in humans and other mammals share up to 95% homology (Tharp and Sarkar, 2013). Eliminating the APP gene has adverse consequences in both animal models and humans. In rodents, knocking out APP does not affect survival, but it disrupts brain development and neurogenesis (Wang et al., 2016). In its soluble state, monomeric Aβ may play beneficial physiological roles in human physiology, such as regulating synaptic activity and antimicrobial effects, and it potentially acts as a vascular “clotting” agent, sealing breaches in the blood–brain barrier (Kumar et al., 2016; Brothers et al., 2018). Furthermore, the plaque burden in the AD brain correlates poorly with disease status and does not reflect clinical phenotype well (Arnold et al., 1991; Braak and Braak, 1991; Nagy et al., 1999). Compared to the brains of subjects in control groups, increased AβO levels in AD brains are associated with severe cognitive impairment and loss of synaptic markers 37; plasma AβOs are negatively correlated with Aβ_42_ concentrations in cerebrospinal fluid and Mini-Mental State Examination (MMSE) scores (Babapour Mofrad et al., 2021). Multiple clinical studies have suggested that AβOs can serve as biomarkers for AD diagnosis (Georganopoulou et al., 2005; Santos et al., 2008; Xia et al., 2009; Wang et al., 2017). The Osaka mutation of APP (E693 deletion) leads to the accumulation of AβO and AD pathology without the formation of senile plaques (Tomiyama et al., 2008; Nishitsuji et al., 2009; Shimada et al., 2011; Jang et al., 2013). In the absence of plaques, intraneuronal Aβ is associated with cognitive impairment in several APP transgenic models (Billings et al., 2005; Knobloch et al., 2007; Leon et al., 2010; Iulita et al., 2014). Importantly, AβO-selective antibodies can prevent AD-like pathology in mice and ameliorates cognitive deficits in AD mice for at least 40 days (Xiao et al., 2013; Zhao et al., 2014; Sebollela et al., 2017; Cline et al., 2018; Viola et al., 2021). The above evidence indicates that AβOs are not only necessary but also sufficient triggers for AD-related neurodegeneration.

The processes by which AβOs cause cellular toxicity and trigger neuronal cell death are divided into three types of toxic effects due to the complex structure and characteristics of AβO as well as the *in vivo* environment (Figure 1).

### 3.1 The physical and chemical interactions between AβOs and neuronal cell membranes

Physical damage to neurons determines neuronal function and survival, both at the neuronal soma and at synapses (Gharahi et al., 2023). As the concentration of AβOs increases, dendritic breakage intensifies, disrupting neuronal cytoskeletal integrity. However, physical collisions or friction alone are insufficient to explain the mechanical damage caused by AβOs to neurons. This is because as Aβ aggregation increases, the bending stiffness and the Young's modulus of the aggregates increase to levels even exceeding those of concrete (Xu et al., 2010); this indicates that these aggregates attain greater structural stability that may result in pathological consequences. This physical damage is partially attributable to the pore-forming effect of AβOs (Bode et al., 2017). AβOs can insert directly into lipid bilayers, altering the structure of the cell membrane and thereby affecting its permeability (Arispe, 2004). AβOs have a greater affinity for membranes than do Aβ monomers, and the barrel-like structure of AβOs allows them to form ion channels within the cell membrane, permitting the entry of Ca^2+^ into the cell. Only the more easily aggregated Aβ_42_ can form ion channels, while Aβ_40_ cannot (Bode et al., 2017). Another possible cause of mechanical damage to neurons is the interaction between AβOs and lipid rafts. Fibrillation of AβOs concentrated on membranes and lipid rafts can lead to membrane rupture and damage, thereby exacerbating Aβ-triggered toxicity (Sciacca et al., 2012). N-methyl-D-aspartic acid receptors (NMDARs) are a class of mechanosensitive receptors, and membrane tension is a major factor that affects NMDAR mechanosensitivity (Singh et al., 2012; Johnson et al., 2019). Although NMDAR activation is a crucial step in memory consolidation, excessive stimulation of NMDARs can lead to Ca^2+^ influx, resulting in neurotoxicity.

In addition to directly damaging the cell membrane, AβOs also bind to membrane-specific receptors, triggering downstream signaling pathways. The potential receptors for AβOs are reviewed in detail by Jarosz-Griffiths et al. (2016). This phenomenon explains why treating cell surfaces with low doses of trypsin almost eliminates AβO binding (Lambert et al., 1998). Accumulating reports have identified many AβO receptors and binding proteins; well-studied AβO receptors include the cellular prion protein (PrPc), which depends on the integrity of cholesterol-rich lipid rafts (Kostylev et al., 2015), and the neuron-specific NaK ATPase α3 subunit (NKAα3), which can lead to neurodegeneration through presynaptic calcium overload (Ohnishi et al., 2015). PrPc binds to AβOs, reducing the NMDAR density at synapses and leading to loss of dendritic spines (Um et al., 2012). PrPc also forms complexes with AβOs, activating metabotropic glutamate receptor 5 (mGluR5) and inducing Ca^2+^ influx. Several other receptors have also been reported to bind directly or indirectly to AβOs, thereby affecting neuronal survival (Table 1).

**Table 1:** Receptors involved in AβOs binding and cytotoxic effects.

**Receptors and membrane proteins**	**Abbreviation**	**References**
Alpha 7-nicotinic acetylcholine receptor	α7nAChR	Dineley et al., 2001; Parri et al., 2011
Alpha-amino-3-hydroxy-5-methylisoxazole-4-propionic acid receptor	AMPA	Zhao et al., 2010; Thomas et al., 2017; Hettinger et al., 2018; Fani et al., 2021
Amylin 3 receptor	AMY3	Fu et al., 2012; Soudy et al., 2019, 2022
Ephrin type A receptor 4	EphA4	Fu et al., 2014; Poppe et al., 2019
Ephrin type B receptor 2	EphB2	Suzuki et al., 2016; Hu et al., 2017a
Immunoglobulin gamma Fc region receptor II-b	FcγRIIb	Kam et al., 2013, 2016; Gwon et al., 2018
Frizzled receptors	FZDs	Magdesian et al., 2008
Glypican 4	Gpc4	Ma et al., 2021
Insulin receptor	IR	Hettinger et al., 2018; Huang et al., 2019; Shigemori et al., 2022; Leclerc et al., 2023
Leukocyte immunoglobulin-like receptor subfamily B member 2/paired Ig-like receptor B	LILRB2/PirB	Hu et al., 2017b; Cao et al., 2018
Metabotropic glutamate receptor 5	mGluR5	Um et al., 2012, 2013
Nogo receptor	NgR	Zhao et al., 2017
NaK ATPase α3 subunit	NKAα3	Ohnishi et al., 2015
Neuroligins	NLs	Brito-Moreira et al., 2017
N-methyl-D-Aspartate receptors	NMDARs	Decker et al., 2010; Back et al., 2021; Fani et al., 2021
p75 neurotrophin receptor	p75NTR	Hashimoto et al., 2004; Andrade-Talavera et al., 2021; Yi et al., 2021; Bruno et al., 2023
Cellular prion protein	PrPc	Kostylev et al., 2015
Receptor of advanced glycation endproducts	RAGE	Takuma et al., 2009; Origlia et al., 2010; Patel and Jhamandas, 2012
Sigma-2/progesterone receptor membrane component 1 Receptors	Sigma-2/PGRMC1 Receptors	Izzo et al., 2014
Transient receptor potential melastatin family subtype 2	TRPM2	Ostapchenko et al., 2015; Li and Jiang, 2018

### 3.2 AβOs and neuronal endocytosis

Endocytosis is a cellular process in which cells engulf extracellular substances. AβOs located in the extracellular space can be internalized into neurons through endocytic vesicles (Takuma et al., 2009; Shi et al., 2020; Teng et al., 2023). Aβ_42_, which forms oligomers more easily than Aβ_40_, binds to the neuronal surface receptor RAGE and is subsequently internalized in a RAGE-dependent manner (Takuma et al., 2009; Seyed Hosseini Fin et al., 2023). In addition, several receptors have been reported to bind to AβOs, allowing AβOs to enter neurons in an endocytic form (Table 1). Aβ accumulates in lysosomal vesicles, which have low internal pH. The rate of assembly of AβOs increases by 8,000-fold when they move from the neutral extracellular environment to the low-pH environment within lysosomes (Schützmann et al., 2021). The acidic environment within endosomes and lysosomes serves as a preferential site for AβO formation (Lee et al., 2022). Aβ-producing sites, such as mitochondria, are also found inside cells (Anandatheerthavarada et al., 2003; Vaillant-Beuchot et al., 2021). A comparison of the damage caused by endocytosis or other transport of Aβ to the direct damage caused by Aβ synthesis within organelles indicates that the latter causes greater damage.

### 3.3 AβO exhibits prion-like spreading effects

After excluding interference from familial AD-related gene mutations, Collinge's research team found, upon autopsy of eight deceased individuals with Creutzfeldt–Jakob disease caused by treatment with human cadaver pituitary-derived growth hormone, that the Creutzfeldt–Jakob disease was caused by the presence of prions in their brains; unexpectedly, abundant Aβ deposits were also found in the gray matter and blood vessels of the brains of four patients (Jaunmuktane et al., 2015). The unique feature of prions lies in their “seeding and spreading” mechanism in which small amounts of misfolded proteins act as seeds which then aggregate and accumulate in the brain, leading to neuronal damage and dysfunction. The results of Collinge et al.'s study suggest that Aβ-related pathology may also occur in humans. Prions can move from one cell to another, thereby spreading abnormal proteins. Similarly, some studies suggest that AβOs can spread from one neuron to another, affecting neighboring cells, and that they can even spread to distant areas through intercellular propagation mechanisms (Ono et al., 2012). Aβ_42_, which is prone to oligomerization, has greater spreading and transfer capability than other Aβ subtypes, and this is partly attributable to the endosomal transport system (Ono et al., 2012).

## 4 Mitochondria are the primary targets of AβOs

The survival of neurons depends on the presence of healthy mitochondria (Cunnane et al., 2020). In addition to being essential for bioenergetics, mitochondria are also involved in neurotoxicity, neuroinflammation, oxidative stress, neural plasticity, and neurodegenerative processes; this places mitochondrial structure and function at the forefront of exploration in various hypotheses related to AD. Mitochondrial abnormalities occurring even before Aβ accumulation, such as during embryonic stages and in young mice, have been observed in animal models exhibiting typical AD symptoms (Naia et al., 2023). More importantly, the colocalization of APP and Aβ with mitochondria provides evidence for the targeted damage of mitochondria by AβOs (Lustbader et al., 2004; Manczak et al., 2006; Wilkins, 2023). Considering the regulatory role of mitochondria in cell fate and energy production, many researchers consider them to be key regulatory factors in neurodegenerative diseases. Interestingly, AβO-induced mitochondrial damage appears to be restricted to neurons and not to occur in astrocytes or microglia (Mastroeni et al., 2018).

## 5 How Aβ accumulates in mitochondria

**Figure 2:**
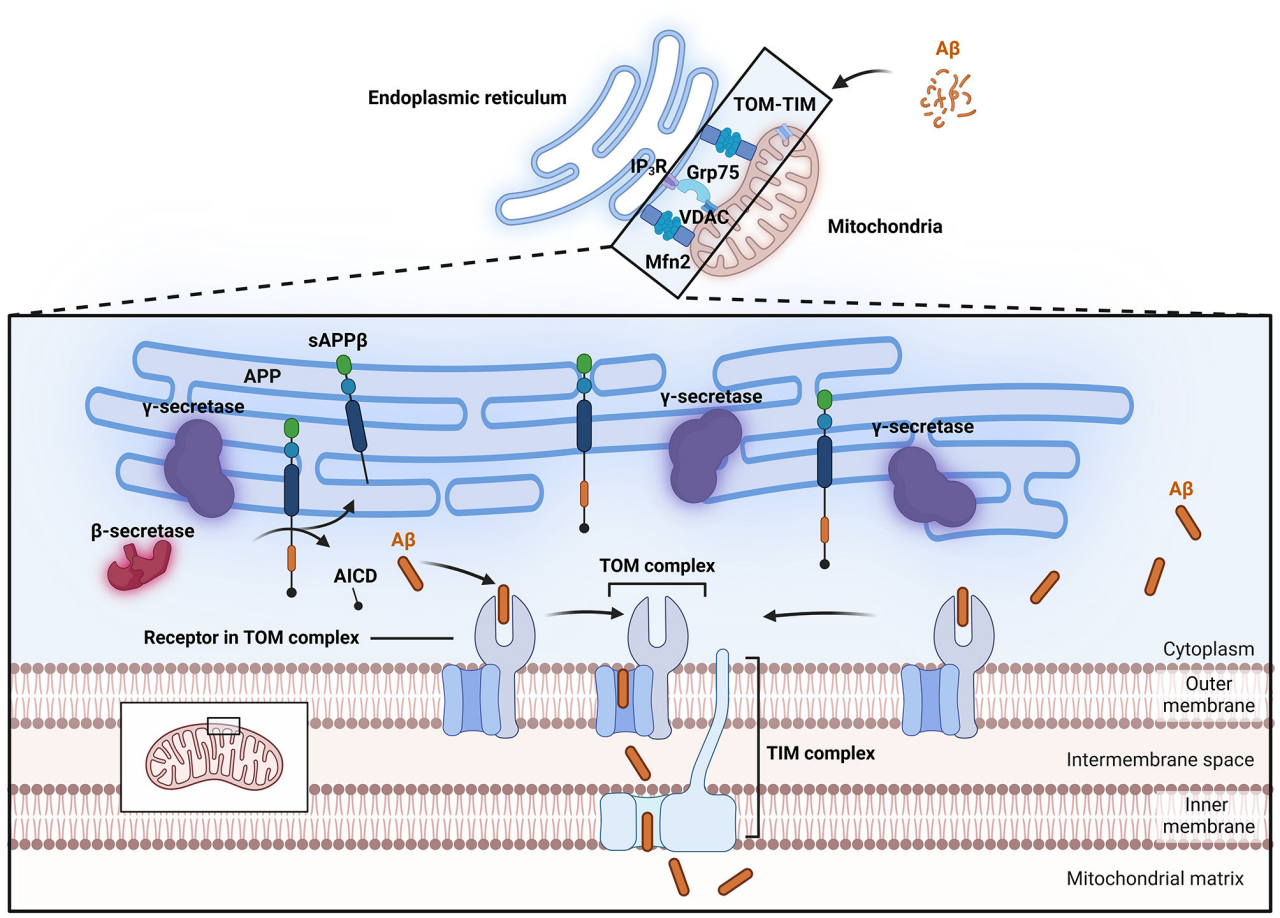
Process of Aβ entry into mitochondria. Aβ can enter mitochondria through the TOM-TIM complex. The MAM has lipid raft properties, and important enzymes involved in APP processing, such as β-secretase and γ-secretase, are located within lipid rafts, allowing APP to be directly cleaved into Aβ and thus enter mitochondria. The close connection of MAMs relies on Mfn2 and Grp75. Grp75 can act as a partner to link IP3R to VDAC. Aβ, β-amyloid protein; AICD, APP intracellular domain; APP, amyloid precursor protein; Grp75, glucose-regulated protein 75; IP_3_R, inositol trisphosphate receptor; MAM, Mitochondria-associated endoplasmic reticulum membranes; Mfn2, mitochondrial fusion protein 2; sAPPβ, soluble APP fragment β; TIM, translocase of the inner membrane; TOM, translocase of the outer membrane; VDAC, voltage-dependent anion channel.

To study the effects of Aβ on mitochondria, it is imperative to study the buildup of Aβ and its physiological effect on mitochondria. Thus, far, at least two distinct pathways of Aβ accumulation in mitochondria have been proposed (Figure 2); the first is the specific translocation of Aβ via translocase of the outer membrane (TOM) and translocase of the inner membrane (TIM) dependent mitochondrial protein transport machinery (Calvo-Rodriguez and Bacskai, 2021; Sayyed and Mahalakshmi, 2022). Sirk et al. (2007) reported that the rates of entry of two endogenous nuclear-encoded mitochondrial proteins, Mortalin/mtHsp70 and TOM20, into mitochondria decreased under conditions of sublethal Aβ_42_ exposure, suggesting that Aβ_42_ occupies the TOM complex, thereby impairing its mitochondrial entry. Research by Hansson Petersen et al. (2008) indicated that Aβ can be internalized and imported into mitochondria via the TOM complex, where it accumulates in mitochondrial cristae. Inhibition of TOM20, TOM70, and TOM40 led to reduced Aβ accumulation in mitochondria. This phenomenon was validated in cortical brain biopsies (Hansson Petersen et al., 2008). Additionally, APP can form complexes with TOM and TIM, leading to accumulation in the mitochondrial import channel (Devi et al., 2006).

Mitochondria-associated endoplasmic reticulum membranes (MAMs) represent another potential pathway for the entry of Aβ into mitochondria. MAMs are contact sites between mitochondria and the endoplasmic reticulum and represent a specialized region where the outer mitochondrial membrane (OMM) is closely associated with the endoplasmic reticulum membrane (Area-Gomez et al., 2018). The close connection between these two organelles relies on mitochondrial fusion protein 2 (Mfn2) and glucose-regulated protein 75 (Grp75; De Brito and Scorrano, 2008; Lee et al., 2019). The physical linkage between the endoplasmic reticulum membrane and the OMM plays crucial roles in lipid synthesis and transport, fatty acid, glucose, and cholesterol metabolism, Ca^2+^ homeostasis, mitochondrial dynamics, and apoptosis. Electron microscopy has shown that the distance between the outer mitochondrial membrane and the endoplasmic reticulum is maintained at ~10–25 nm, preserving the unique structure of these organelles and providing a functional platform for various proteins (Csordás et al., 2006). Clinically, fibroblasts from both familial and sporadic AD patients exhibit upregulated MAM function, indicating increased interaction between the endoplasmic reticulum and mitochondria (Area-Gomez et al., 2012). Further research in this area indicated that exposure to Aβ that is in a low oligomeric state increases the number of MAM contact points in SH-SY5Y cells, leading to enhanced Ca^2+^ transfer between organelles (Hedskog et al., 2013). Other studies suggest that the C99 fragment is highly enriched in subcompartments of the endoplasmic reticulum associated with mitochondria (Pera et al., 2017; Montesinos et al., 2020). Schon's laboratory also reported that two important subunits of the catalytic presenilin subunit of γ-secretase, presenilin-1 (PS1) and presenilin-2 (PS2), are highly enriched in MAMs (Area-Gomez et al., 2009). MAMs also exhibit lipid raft characteristics, thus providing a favorable environment in which γ-secretase activity can target APP (Li et al., 2023b). Important enzymes involved in APP processing, such as β- and γ-secretases, are located within lipid rafts. Changes in the cholesterol and sphingolipid content of the cell membrane or alterations in the distribution of cholesterol within lipid rafts can disrupt the structure and function of the rafts, thereby altering the activity of β- and γ-secretases, increasing their contact and interaction with APP, and ultimately leading to increased generation and deposition of Aβ in the brain (Del Prete et al., 2017; Sanderson, 2022). These observations support the idea that the MAM is a potential site for Aβ production near mitochondria and indicate that this pathway may be a possible source of the Aβ localized within mitochondria.

## 6 The damage caused by AβOs to mitochondria

**Figure 3:**
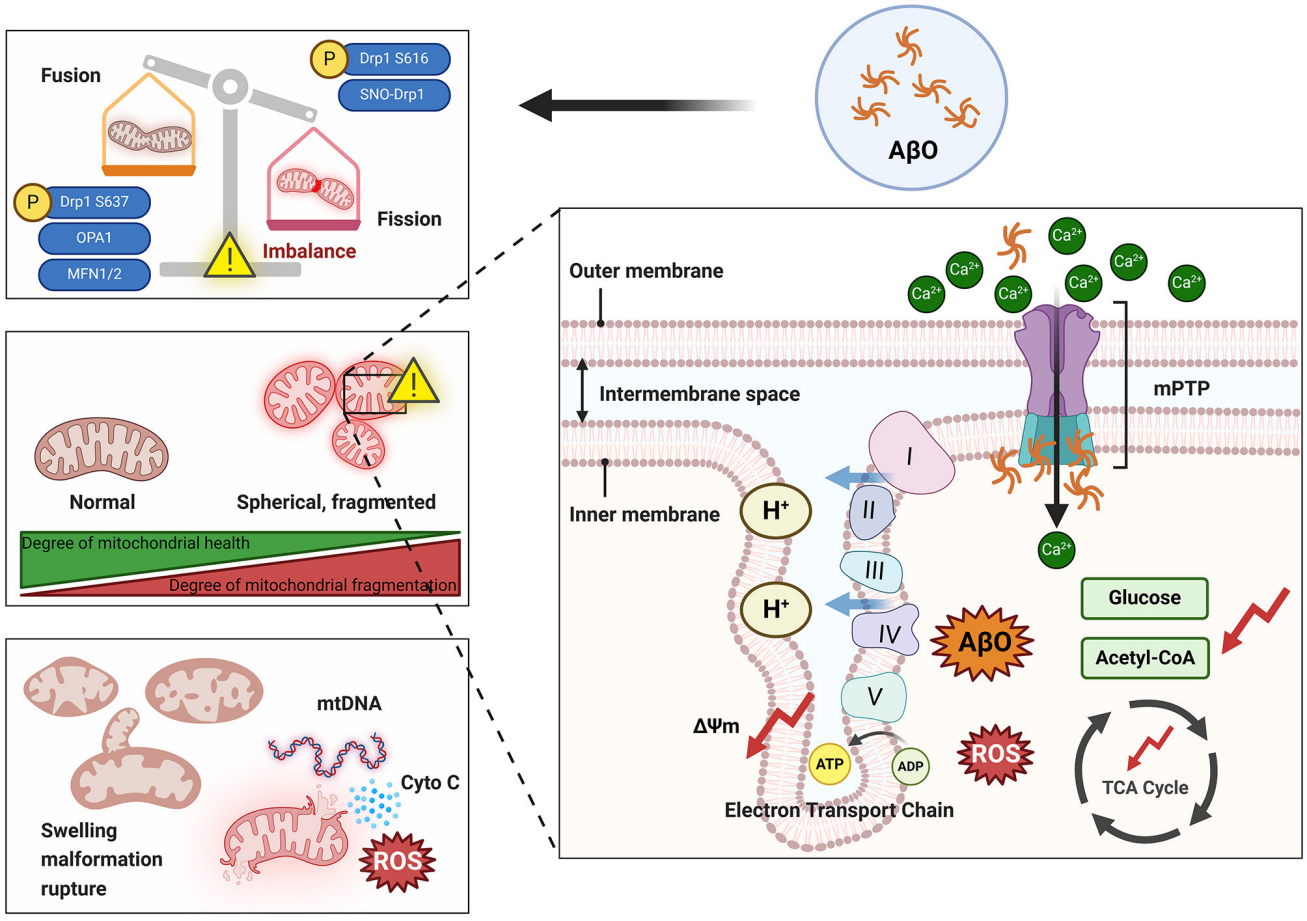
Damage to mitochondria by AβO. The presence of AβOs can lead to an imbalance in mitochondrial dynamics that is manifested by increased mitochondrial fission and overfusion and increased mitochondrial fragmentation. Overfragmented mitochondria exhibit swelling and spherical morphology that damages the cristae structure. Additionally, AβOs can activate the mPTP, leading to a large influx of Ca^2+^ into mitochondria and resulting in increased production and accumulation of ROS and inhibition of the TCA cycle. Ultimately, mitochondria rupture and release their contents into the cytoplasm. ΔΨm, Mitochondrial membrane potential; AβO, Aβ oligomer; ADP, adenosine diphosphate; ATP, adenosine triphosphate; Cyto C, cytochrome C; Drp1, dynamin-related protein 1; Mfn1, mitochondrial fusion protein 1; Mfn2, mitochondrial fusion protein 2; mPTP, mitochondrial permeability transition pore; mtDNA, mitochondrial DNA; OPA1, optic atrophy 1; ROS, reactive oxygen species; SNO, S-nitrosylation; TCA, tricarboxylic acid. I, Complex I; II, Complex II; III, Complex III; IV, Complex IV; V, Complex V.

Mitochondrial dysfunction is one of the primary markers of neuronal toxicity induced by AβO in the pathogenesis of AD. Aβ and its oligomers have been reported to participate in mitochondrial dysfunction through various mechanisms (Peng et al., 2020). These mechanisms include disruption of mitochondrial morphology (e.g., through damage to mitochondrial cristae), interference with normal electron transport chain processes, induction of oxidative stress, and impairment of mitochondrial dynamics, resulting in disruption of the normal processes of mitochondrial fission and fusion. These disruptions can impair ATP production, increase oxidative damage, decrease mitochondrial self-repair, and trigger neuronal apoptosis (Figure 3). Understanding the complex relationship between AβO and mitochondrial damage is crucial for elucidating the molecular mechanisms that underlie AD and for developing targeted therapeutic strategies.

### 6.1 The disruption of the balance of mitochondrial dynamics caused by AβOs

Underlying the general stability of the mitochondrial network structure are the dynamic processes of mitochondrial fission and fusion (Huang et al., 2023). The balance between fusion and fission processes in mitochondria is dependent on several proteins that belong to the guanosine triphosphatase (GTPase) family. In mammalian cells, the mitochondrial dynamics proteins MiD 49 (a 49-kDa protein) and MiD51, fission protein 1 (Fis1), mitochondrial fission factor (Mff), and dynamin-related protein 1 (Drp1) have been identified as the main regulators of mitochondrial fission in mammals (Chen et al., 2023). During the process of mitochondrial fusion, two dynamin-related proteins, namely, optic atrophy 1 (OPA1) and Mfn1/2, induce OMM fusion, and OPA1 also mediates inner mitochondrial membrane (IMM) fusion (Van Der Bliek et al., 2013).

Disruption of mitochondrial dynamics homeostasis, including mitochondrial overfusion or fission, is detrimental to neuronal survival (Flippo and Strack, 2017). Fragmented mitochondria (increased fission) and reduced fusion, resulting in the formation of smaller, dysfunctional mitochondria with abnormal cristae, have been observed in human AD patients (Baloyannis, 2006; Calkins et al., 2011). Furthermore, treating SH-SY5Y cells with AβO, overexpressing APP in N2a cells, or culturing fibroblasts from patients with sporadic and familial AD resulted in excessive activation of Drp1 and reduced expression of Mfn1/2. This effect manifests as abnormal Drp1 accumulation around the nucleus and an increase in Drp1 binding to Fis1, leading to excessive mitochondrial fragmentation, functional impairment, and cellular toxicity (Wang et al., 2008a; Park et al., 2015; Joshi et al., 2018; Ahmed et al., 2019). Detection of changes in the expression of genes related to mitochondrial dynamics contributes to the early diagnosis of mild cognitive impairment (MCI; Liu et al., 2023). Reddy's laboratory observed increased expression of Drp1 and Fis1 in the frontal cortices of patients at Braak stages I and II (early AD), III and IV (definite AD), and V and VI (severe AD) compared to controls, whereas the expression of Mfn1, Mfn2, OPA1, and TOM40 decreased. Furthermore, Drp1 interacts with AβO in Braak stages I and II, III and IV, and V and VI, and the intensity of this interaction increases as AD progresses (Manczak et al., 2011). In primary neurons obtained from AD model mice (Tg73.7 line), *Calkins* and colleagues used dual labeling with A11 and the mitochondrial matrix marker cyclophilin D (CypD) to demonstrate colocalization of AβO with CypD and accumulation of AβO; these changes resulted in reduced mitochondrial anterograde transport, increased mitochondrial fission and decreased mitochondrial fusion, and electron microscopy revealed that numerous mitochondria lacked clear membrane structures and exhibited fragmentation (Calkins et al., 2011). Over time, AβO was found to gradually accumulate in neuronal processes and cell bodies, consistent with previously obtained clinical data. Drp1 and Fis1 can bind to receptors on the OMM after various posttranslational modifications (including S-nitrosylation, phosphorylation, SUMOylation, dephosphorylation, and ubiquitination) and thereby promote or inhibit mitochondrial fission. S-nitrosylation of Drp1 mediated by AβO forms SNO-Drp1 and leads to excessive mitochondrial fragmentation, a severe deficit in bioenergetics, and neuronal abnormalities (Cho et al., 2009). Phosphorylation is the most common posttranslational modification of Drp1. Drp1 has two main phosphorylation sites, Ser616 and Ser637; phosphorylation at these sites induces and inhibits mitochondrial fission, respectively (Chou et al., 2012). Drp1 phosphorylated at Ser616 translocates to the mitochondrial outer membrane, where it exhibits increased GTPase activity and causes mitochondrial constriction and fragmentation. Soluble AβO inhibits excessive phosphorylation of Drp1 at Ser637 in primary rat neurons, and this significantly increases mitochondrial fission in the dendrites of these neurons (Sarkar et al., 2015). Based on studies conducted in the three cell lines SK-N-MC, SK-N-SH, and SH-SY5Y, Kim et al. (2016) suggested that AβO activates Akt, directly induces phosphorylation of Drp1 at Ser616, promotes mitochondrial fragmentation, and triggers downstream events such as the release of reactive oxygen species (ROS). AβO can also disrupt neuronal antioxidant function, leading to ROS accumulation and inhibition of Mfn1/2 expression (Park et al., 2015). In regulating mitochondrial fusion, OPA1 depends on Mfn1 (Cipolat et al., 2004). The expression of OPA1 is downregulated by cellular prion protein, leading to depletion of mtDNA and neuronal apoptosis, while overexpression of OPA1 can partially reverse cellular apoptosis induced by prion protein (Wu et al., 2019). Moreover, overexpression of Mfn2 rather than Mfn1 significantly inhibits the AβO-mediated cell death pathway (Park et al., 2015). *In vivo* and *in vitro* evidence indicates that inhibiting Drp1-induced mitochondrial fission can protect against synaptic injury in AD model mice and N2a cells (Baek et al., 2017; Reddy et al., 2017); heterozygous Drp1 knockout mice exhibit significantly reduced levels of AβO (Manczak et al., 2012). The presence of the Drp1-S579A mutant, which has a phosphorylation defect at the Ser579 site, reduces apoptosis in primary mouse neurons treated with Aβ_42_, emphasizing the potential of targeting Drp1 to alleviate Aβ neurotoxicity (Xu et al., 2021a). Furthermore, N2a neurons treated with the mitochondrial activators MitoQ and SS-31 exhibit more intact mitochondrial structures than do N2a cells coincubated with AβO (Aβ_25 − 35_), showing that these activators reverse the AβO-induced imbalance in mitochondrial dynamics (Manczak et al., 2010).

### 6.2 The damage to mitochondrial membrane permeability and mitochondrial cristae caused by AβOs

The mechanism through which mitochondrial damage occurs is closely related to pathological changes in mitochondrial ultrastructure. As organelles enveloped by two membranes, mitochondria can be divided into four functional regions; from inside to outside, these are the matrix, the IMM, the intermembrane space, and the OMM (Cogliati et al., 2016). The mitochondrial permeability transition pore (mPTP), also known as the mitochondrial channel, is a channel between the inner and outer mitochondrial membranes that is composed of proteins. The mPTP is the basis for permeability transition; it consists mainly of the protein CypD, which is located in the matrix, the voltage-dependent anion channel (VDAC) located in the outer membrane, and the adenine nucleotide translocator (ANT) in the inner membrane (Jahn et al., 2023). Like most pore-forming toxins, AβO can induce perforation of neuronal and mitochondrial membranes; this is considered a mechanism of neurotoxicity induction and is observed in human AD patients (Lin et al., 2001; Lashuel et al., 2002, 2003; Inoue, 2008; Tong et al., 2018). Oligomeric forms of Aβ facilitate opening of the mPTP, which results in activation of a mitochondria-dependent cascade of neuronal cell death (Arrázola et al., 2017). In AD mouse models and *in vitro*, AβO interacts directly with CypD, forming specific complexes, inducing mPTP activation, and causing an imbalance in membrane permeability (Moreira et al., 2001; Shevtzova et al., 2001). The VDAC has Ca^2+^-binding sites that regulate its permeability, and abnormally high Ca^2+^ concentrations can open the mPTP (Kuchibhotla et al., 2008). Recent studies have suggested that AβO can disrupt membranes and create conductive pores, potentially disrupting cellular Ca^2+^ homeostasis and driving neurotoxicity in AD (Li et al., 2023a). The Ca^2+^ concentration is strictly controlled under physiological conditions, but abnormal increases in Ca^2+^ concentration have been observed during AD development (Calvo-Rodríguez et al., 2016; Calvo-Rodriguez et al., 2020). Due to the self-aggregation properties of Aβ, the concentration of Ca^2+^ in the spines and dendrites of surrounding cortical pyramidal neurons is greater than normal in adjacent resting neurons, and Ca^2+^ selectively accelerates the formation of Aβ_42_ fibrils (Ahmad et al., 2009; Tong et al., 2018). Aβ deposition is a necessary condition for triggering neuronal mitochondrial Ca^2+^ overload in AD (Calvo-Rodriguez et al., 2020). When the intracellular Ca^2+^ concentration increases, mitochondria rapidly take up Ca^2+^ to prevent Ca^2+^ overload in the cytoplasm. However, entry of excessive Ca^2+^ into mitochondria leads to mitochondrial Ca^2+^ overload, increased ROS production, inhibition of ATP synthesis, opening of mPTPs, and the subsequent release of cytochrome C (Cyto C) and activation of apoptotic pathways (Bernardi, 1999). The dual stimulation by Ca^2+^ and ROS further increases mitochondrial membrane permeability, leading to membrane depolarization and mitochondrial swelling. Indeed, APP/PS1 mouse models exhibit abnormal mitochondrial structure, including mitochondrial swelling and fragmentation (Xie et al., 2013). Treatment with Anle138b (an Aβ channel blocker) can eliminate Ca^2+^ oscillations in aged 3xTg mice (Li et al., 2023a).

ROS mainly appear as chemical substances that are active in the aerobic metabolic processes of cells. Metal ions can bind to Aβ and participate in the production of ROS originating from non-mitochondrial sources; for details on this process, one can refer to the excellent review by Cheignon et al. (2018). ROS are gradually released from mitochondria into the cytoplasm, passing through the OMM in a process that requires mediation by VDAC (Brdiczka et al., 2006). VDAC colocalizes with APP and AβO in the frontal cortex of AD patients, confirming its relevance to AD progression (Manczak and Reddy, 2012). The mitochondrial double-membrane pore has two oxidation-sensitive sites. One of these sites has a pyridine nucleotide-binding motif that can bind to nicotinamide adenine dinucleotide (NAD) and nicotinamide adenine dinucleotide phosphate (NADPH); the other site has a glutathione-binding motif that can bind to glutathione (Bernardi et al., 2023). ROS can oxidize NADH, NADPH, and glutathione bound to mitochondrial double-membrane pores and thereby promote the opening of these membrane pores. Treatment with antioxidants can help cells restore the appropriate ROS balance and reduce the damage caused by AβO to the mitochondrial double membrane; such treatment shows promising therapeutic effects in patients with MCI and in animal models of AD (Peng et al., 2020; Sousa et al., 2023).

The folding of the IMM creates the mitochondrial cristae, which can be described as a structure made up of elementary particles, the intracristae space, and the intercristae space. The elementary particles in the cristae are arranged perpendicular to the membrane surface and house ATP synthase enzymes capable of converting ADP to ATP using the energy released during electron transfer in the respiratory chain (Friedman and Nunnari, 2014). Autopsy reports on AD patients indicate that there is a significant decrease in the proportion of normal mitochondria and a marked increase in the proportion of fractured cristae in their neurons compared to those of controls (Hirai et al., 2001). In addition, severe disruption of the mitochondrial cristae in N2a cells following exposure to AβOs has been observed via electron microscopy (Manczak et al., 2010). The mitochondrial inner membrane protein MIC60 densely fills the connections between cristae, while OPA1 is localized to the cristae membrane and the inner boundary membrane of the mitochondrion, suggesting that it mediates the formation of new cristae by directly folding the inner mitochondrial membrane (Barrera et al., 2016). However, there is insufficient direct evidence linking AβOs to mitochondrial cristae damage. Failure to maintain normal mitochondrial morphology is detrimental to the bioenergetic activity of mitochondria (Kondadi et al., 2019). The electron transport chain complexes (complexes I–V) of the mitochondrial electron respiratory chain are crucial links between cristae morphology and mitochondrial function and are embedded in the IMM (Cogliati et al., 2013). The presence of cristae increases the surface area of the mitochondrial inner membrane, making aerobic respiration more efficient. Disruption of the structure of the cristae can directly lead to deformities in ETC complexes, disrupting electron transfer and proton transport and ultimately resulting in electron and proton leakage, reduced ATP production, and increased ROS generation and necessitating the restoration of cristae structure to maintain normal ETC function (Lin and Beal, 2006). Several points that are relevant to this are worth considering: AβOs challenge the equilibrium of mitochondrial dynamics, and an imbalance in mitochondrial dynamics directly affects cristae structure. For instance, OPA1 is a key regulator of mitochondrial cristae remodeling, and excessive mitochondrial fission can promote disruptions in cristae structure (Frezza et al., 2006; Anand et al., 2014; Otera et al., 2016; Del Dotto et al., 2017). Additionally, embryonic fibroblasts from Mfn1/2 double knockout mice contain numerous fragmented mitochondria. These morphological changes in mitochondria lead to a decrease in the rate of cell growth, loss of mitochondrial membrane potential and heterogeneity, and reduced respiration, indicating that mitochondrial OXPHOS is impaired when mitochondria are fragmented (Chen et al., 2005).

### 6.3 Effect of AβOs on mitochondrial respiratory chain function and energy metabolism

Neurons are highly energy-demanding cells that rely heavily on OXPHOS systems (Ronowska et al., 2018). As the cell's energy generators, mitochondria produce ATP through OXPHOS and utilize the metabolites of the tricarboxylic acid (TCA) cycle to support macromolecular synthesis (Area-Gomez et al., 2019; Arnold and Finley, 2023). The TCA cycle is a system of enzyme-catalyzed reactions that oxidize acetyl coenzyme A (CoA) to CO_2_. OXPHOS is the coupling mechanism between electron transport and ATP formation. Mitochondria produce ATP and metabolites from a variety of carbon fuels, including acetate, glutamine, and amino acids produced through the glycolytic pathway. CoA derived from acetyl-CoA, amino acid, or fatty acid oxidation is directed into reaction cycles that sustain energy production, synthetic metabolism, catabolic processes, and redox balance. In AD, abnormalities in the activities of mitochondrial enzymes involved in energy production, such as the pyruvate dehydrogenase (PDH) complex, citrate synthase, isocitrate dehydrogenase, and α-ketoglutarate dehydrogenase (αKGDH), impair the maintenance of mitochondrial membrane potential, leading to decreased ATP production (Sheu et al., 1994; Gibson et al., 1998; Pagani and Eckert, 2011; Beck et al., 2016; Cenini and Voos, 2019; Chhimpa et al., 2023; Andreyev et al., 2024).

Glutamate, an amino acid that can serve as a neurotransmitter, is synthesized from the TCA cycle intermediate α-ketoglutarate (αKG). Glutamate metabolism is crucial for metabolism and signaling in neurons and glial cells (Mckenna et al., 2016). Therefore, it can be inferred that defective mitochondrial bioenergetics may lead to impaired neuronal activity and synaptic transmission. Glucose is the primary energy substrate in the brain, as it is a major source of ATP. Reduced glucose uptake and decreased TCA activity are typical features of AD. Using brain imaging methods, neurofibrillary tangles can be detected in brain regions that show impaired glucose uptake (Hoyer et al., 1988; Kalaria and Harik, 1989; Jagust et al., 1991). AβOs can bind to neuronal insulin receptor (IR), triggering downstream phosphorylation of Akt at Ser473 and leading to brain insulin resistance and reduced neuronal glucose uptake (Zhao et al., 2008). Hence, AD is also referred to as “type 3 diabetes” (Arnold et al., 2018; Leclerc et al., 2023). AβOs also impair glucose uptake in cortical and hippocampal neurons via 4-hydroxynonenal, reducing ATP levels and leading to glucose transporter 3 (GLUT3) binding and lipid peroxidation (Mark et al., 1997). In a low ATP state, mitochondria are unable to pump Ca^2+^ from the IMM, and the resulting accumulation of Ca^2+^ causes mitochondrial dysfunction (Calvo-Rodriguez and Bacskai, 2021). Neurons absorb glucose via high-affinity GLUT3 transporters and convert it to pyruvate in the glycolytic pathway. Pyruvate enters mitochondria via the pyruvate carrier and is metabolized to CoA by the PDH complex in the mitochondrial matrix. The first step in the TCA cycle is the condensation of CoA with oxaloacetate to form citrate; this reaction is catalyzed by citrate synthase, which drives the reaction by hydrolysis of the high-energy thioester bond of CoA. Despite the lack of evidence that AβOs directly affect the enzymes of the TCA cycle, some *in vitro* experiments have suggested that addition of Aβ to the cell culture medium of primary and cloned neurons and glial cells inhibits key enzymes of the PDH complex and the TCA cycle (Bielarczyk et al., 2003, 2006; Szutowicz et al., 2005, 2006). Additionally, Aβ affects tau protein kinase 1 (TPK1), which, by over phosphorylating tau protein, inhibits PDH, thereby inhibiting mitochondrial reactions involving acetyl-CoA and citrate, reducing acetylcholine synthesis, and leading to memory and cognitive impairments (Ihara et al., 1986; Arriagada et al., 1992; Hoshi et al., 1996).

The respiratory chain enzyme complex located within the IMM consists primarily of five enzyme complexes. These complexes oxidize the reductive NADH and succinate generated during glycolysis, the TCA cycle, and β-oxidation, producing NAD^+^ and NADP^+^ while pumping protons out of the mitochondria (Area-Gomez et al., 2019).

The AβOs imported into mitochondria by TOM localize and aggregate near the cristae of the IMM, which is the most active region for OXPHOS (Hansson Petersen et al., 2008). Failure to clear AβOs from the matrix in a timely manner, which can occur due to mutations in the mitochondrial matrix enzyme pitrilysin metallopeptidase 1 (PITRM1) or to reduced human presequence protease (PreP) activity, leads to a severe decrease in OXPHOS (Fang et al., 2015; Brunetti et al., 2016). AβO treatment did not harm ρ0 cells, validating the idea that the harmful effects of AβOs on mitochondria occur through direct or indirect interactions with the ETC (Swerdlow, 2020; Stojakovic et al., 2021). Damage to the ETC by AβOs is the primary cause of OXPHOS decline, and Complex IV has been extensively studied because AβOs appear to specifically inhibit this process (Canevari et al., 1999; Parks et al., 2001). The laboratories of Reddy and Behbahani both observed defects in the activity of Complex IV in mitochondria in the brains of transgenic mouse models expressing human APP (Manczak et al., 2006; Rönnbäck et al., 2016). Preaggregated Aβ_42_ inhibited the activity of isolated Complex IV, while monomeric Aβ did not exhibit toxicity (Crouch et al., 2005, 2006); this may be partially attributed to the presence of free Cu^2+^ ions in cells. Such ions can form metal complexes with Aβ, promote Aβ oligomerization, and potentially interact with Complex IV through a hemin-Cu-mediated dioxygen reduction reaction (Atwood et al., 1998; Klug et al., 2003; Branch et al., 2017; Adam et al., 2018). The amino acid sequence of Aβ typically resides in the heme-binding pocket of hemoglobin. Hemoglobin has been shown to be deficient in AD (Bush, 2003; Shah et al., 2011). Complex IV consists of a class of electron transfer proteins that use heme (or heme iron) as cofactors. AβOs can bind to heme to form peroxidases, forms a peroxidase active site, inhibiting further aggregation of Aβ and breaking down AβOs (Atamna and Boyle, 2006). In addition, Aβ can form a complex with Aβ-binding alcohol dehydrogenase (ABAD) and inhibit NAD production, leading to a decrease in Complex IV activity, affecting electron transfer, causing ROS accumulation, reducing ATP levels, and ultimately affecting mitochondrial energy metabolism (Lustbader et al., 2004). Like Complex IV, Complex V has also been shown to be defective in AD (Schmidt et al., 2008). Oligomycin sensitivity-conferring protein (OSCP), a subunit of Complex V, is sensitive to oligomycin (Giorgio et al., 2019). The physical interaction between OSCP and Aβ results in the selective loss of OSCP subunits and decreased Complex V function, and this leads to reduced ATP production, increased ROS production, and mitochondrial calcium-induced mPTP activation (Beck et al., 2016). Other researchers compared the levels of major F1FO-ATP synthase subunits in the brains of AD individuals, individuals with MCI, and non-AD control subjects and found a selective decrease in OSCP levels during AD progression (Beck et al., 2016). Notably, one of the important factors in the formation of mitochondrial cristae is the dimerization of Complex V. Dimerization of ATP synthase contributes to the formation and preservation of normal cristae and to an increase in OXPHOS performance (Strauss et al., 2008; Habersetzer et al., 2013; Cogliati et al., 2016). In addition to Complex IV and ATP synthase, PreP in the mitochondrial matrix also affects OXPHOS. Accumulation of Aβ in mitochondria reduces PreP activity, triggering peptidase inactivation and the accumulation of unprocessed proteins; this, in turn, imposes feedback inhibition on normal targeting peptide processing and mitochondrial protein degradation and causes imbalances in the mitochondrial proteome (Mossmann et al., 2014). Interestingly, analysis of mitochondrial proteomes in the synaptosomal and non-synaptosomal fractions of AD mice revealed significant changes in the content of proteins related to OXPHOS, suggesting that changes in protein homeostasis may play a role in impairing OXPHOS function (Völgyi et al., 2017). In addition to its effect on Complexes IV and V, exogenous Aβ can also reduce the activity of ETC Complexes I and III in PC12 cells and transgenic animals (Rhein et al., 2009b; Monteiro-Cardoso et al., 2015; Martino Adami et al., 2017; Stojakovic et al., 2021). Rhein et al. (2009a) observed a direct inhibitory effect of monomeric and oligomeric forms of extracellular Aβ on the interaction among Complex I subunits. However, recent studies have reached different conclusions. When AβOs accumulate, dephosphorylation of the evolutionarily conserved signaling intermediate of the mitochondrial assembly factor complex core protein in the Toll pathway becomes more pronounced, suggesting excessive assembly of Complex I that results in ROS overproduction and ultimately in damage to the respiratory chain (Mcgregor et al., 2023).

## 7 Apoptosis, pyroptosis, and necroptosis

Cell death is a highly regulated and critically important homeostatic mechanism that is necessary for maintaining tissue and organ size and function. Mature neurons are terminally differentiated cells with limited proliferative or replacement capacity. Consequently, neuronal death is particularly important both in development and in pathological processes. PCD, also known as apoptosis, is a mechanism by which cells can self-destruct in a controlled manner. PCD plays a crucial role in various biological processes, including embryonic development, tissue homeostasis, and the immune system (Galluzzi et al., 2018). Apoptosis, pyroptosis, and necroptosis are the types of PCD that have been the most studied. The essential molecules involved in these PCD pathways have been successfully identified, and the fundamental roles of PCD in a variety of disorders, including cancer, infectious diseases, inflammatory diseases, and normal development, have been methodically clarified (Vitale et al., 2023).

Cell apoptosis is initiated by caspase-8, caspase-9, and caspase-10 and executed by caspase-3, caspase-6, and caspase-7(Vitale et al., 2023). Pyroptosis is mediated by large protein complexes called inflammasomes, which are activated upon the detection of pathogen-associated molecular patterns (PAMPs) or danger-associated molecular patterns (DAMPs). Activated inflammasomes further activate caspase-1 and caspase-11 (in mice) or caspase-4/5 (in humans); these caspases cleave gasdermin D (GSDMD), releasing its N-terminal fragment. This fragment forms pores in the cell membrane that lead to cell swelling and osmotic lysis (Han et al., 2020). Moreover, the N-terminal fragment of GSDMD can trigger an inflammatory response by promoting the release of the proinflammatory cytokines interleukin (IL)-1β and IL-18. In necroptosis, receptor-interacting protein kinase 1 (RIPK1) and RIPK3 play central roles (Molnár et al., 2019). Mixed lineage kinase domain-like protein (MLKL) is a downstream effector molecule of necroptosis. Phosphorylation of MLKL at Ser227 by RIPK3 further phosphorylates and oligomerizes MLKL, exposing its N-terminal domain and causing it to translocate to the plasma membrane, where it binds to and permeabilizes the membrane. This leads to disruption of membrane integrity, cell swelling, and ultimately cell lysis. Although the pathways leading to caspase activation in these three forms of PCD have traditionally been considered independent, increasing evidence suggests that there is significant crosstalk among these pathways, especially in neuronal cells (Fricker et al., 2018). The complexity of the crosstalk among the PCD pathways implies the existence of dynamic molecular interaction networks rather than isolated pathways guiding cell death events. These data have led to the emergence of the concept of PANoptosis (“P,” pyroptosis; “A,” apoptosis; “N,” necroptosis; and “optosis”) as a way to define a unique, physiologically relevant form of PCD that is activated by specific triggering factors and exhibits molecular characteristics of pyroptosis, apoptosis, and/or necroptosis that cannot be explained by any one of these PCD pathways alone (Wang and Kanneganti, 2021). PANoptosis was first reported in 2016 (Kuriakose et al., 2016). Macrophages infected with influenza A virus (IAV) undergo cell death in a manner that is similar to PANoptosis; it is characterized by the activation of caspase-1, caspase-8, and caspase-3 and the phosphorylation of MLKL, which are fundamental molecular events of pyroptosis, apoptosis, and necroptosis (Kuriakose et al., 2016). The diverse modes of cell death that exist make the identification and evaluation of the impact of unique cell death mechanisms in neurodegenerative diseases both critical and challenging.

## 8 PANoptosis and its initiation

**Figure 4:**
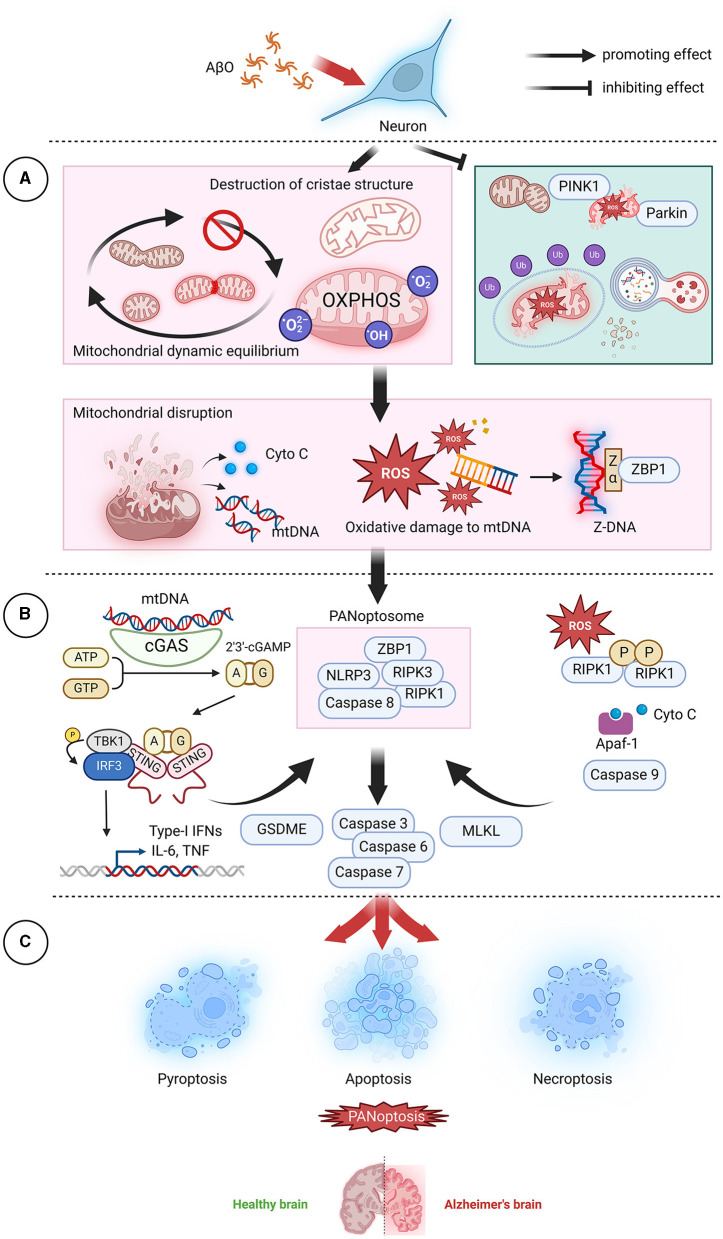
AβO-induced mitochondrial dysfunction leads to PANoptosis. **(A)** In neurons, AβOs induce mitochondrial dysfunction and inhibit mitochondrial autophagy, leading to the release of mitochondrial contents, which act as the top sensors of PANoptosis. **(B)** Cyto C can bind to Apaf-1 and activate caspase 9, subsequently activating the downstream effectors caspase 7 and caspase 3 and initiating apoptosis. mtDNA, a DAMP, can be recognized and transduced by cGAS-STING and NLRP3, resulting in promotion of the assembly of PANoptosomes and initiation of pyroptotic signaling pathways. ROS can promote RIPK1 autophosphorylation and thereby facilitate the formation of necroptotic bodies and the activation of RIPK1, RIPK3, and MLKL. Additionally, ROS cause oxidative damage to mtDNA, generate Z-DNA that is recognized by ZBP1, cause the assembly of PANoptosomes, and activate PANoptosis. **(C)** In the brain, neuronal cell death via PANoptosis may be the underlying factor leading to the development of AD. AβO, Aβ oligomer; AD, Alzheimer's disease; Apaf-1, Apoptotic protease activating factor-1; ATP, adenosine triphosphate; cGAMP, Cyclic GMP-AMPP; cGAS, Cyclic GMP-AMP Synthase; Cyto C, cytochrome C; DAMP, danger-associated molecular pattern; GSDME, Gasdermin E; GTP, guanosine triphosphate; IFN, interferon;IL-6, interleukin- 6; MLKL, mixed lineage kinase domain-like protein; mtDNA, mitochondrial DNA; NLRP3, NOD-like receptor thermal protein domain associated protein 3; OXPHOS, oxidative phosphorylation; PINK1, PTEN-induced kinase; RIPK1, receptor-interacting protein kinase 1; RIPK3, receptor-interacting protein kinase 3; ROS, reactive oxygen species; STING, stimulator of interferon gene; TNF, tumor necrosis factor; ZBP1, Z-DNA binding protein 1.

PANoptosis involves a complex called the PANoptosome, which serves as a platform for key molecules involved in pyroptosis, apoptosis, and/or necroptosis. Accumulating evidence suggests that PANoptosis almost always involves the assembly of a multiprotein complex, the PANoptosome, which serves as a platform for key molecules involved in pyroptosis, apoptosis, and/or necroptosis and is critical for initiating cell death and sensing PAMPs, DAMPs, and other danger factors (Pandian and Kanneganti, 2022; Sharma et al., 2023). The composition of the PANoptosome varies depending on the triggering factor, but almost all PANoptosomes contain Z-DNA-binding protein 1 (ZBP1), absent in melanoma 2 (AIM2), RIPK3, RIPK1, apoptosis-associated speck-like protein (ASC), which contains a caspase recruitment domain, Fas-associated death domain protein (FADD), caspase 8, and key components that execute pyroptosis, apoptosis, and necroptosis (Wang and Kanneganti, 2021; Gullett et al., 2022; Shi et al., 2023a; Zhu et al., 2023). Thus, the PANoptosome has become a focal point and an entry point for studying and regulating PANoptosis (Figure 4). To date, two upstream molecules, ZBP1 and RIPK1, that respond to specific stimuli in a way that triggers PANoptosome assembly have been identified. ZBP1 is a Z-DNA recognition protein containing two Zα domains that are capable of binding Z-DNA/Z-RNA and two RIP homotypic interaction motif (RHIM) domains that interact with other RHIMs in RIPK1 and RIPK3 (Schwartz et al., 2001; Wang et al., 2008b; Kesavardhana et al., 2020; Muendlein et al., 2021). The ZBP1-PANoptosome induces cell pyroptosis, apoptosis, and necroptosis by activating the NOD-like receptor thermal protein domain-associated protein 3 (NLRP3)/ASC/caspase 1-GSDMD and caspase 3/6/7 and MLKL pathways, ultimately triggering PANoptosis (Zheng et al., 2020). The loss of ZBP1 is sufficient to completely inhibit PANoptosis, while the loss of individual components of typical PCD pathways cannot rescue cell death (Kesavardhana et al., 2020). RIPK1 contains an N-terminal kinase domain, a C-terminal death domain, and an intermediate domain containing RHIM. The PANoptosome assembled by RIPK1 forms during *Yersiniavan* infection; the apoptotic and pyroptotic arms of this complex require RIPK1, while the necroptosis arm is inhibited by RIPK1, suggesting that the PANoptosome can differentially regulate the effectors of pyroptosis, apoptosis, and necroptosis (Malireddi et al., 2020a). In contrast to PANoptosome-initiated PANoptosis, transforming growth factor β-activated kinase 1 (TAK1) inhibits the initiation of PANoptosis (Orning et al., 2018; Sarhan et al., 2018; Malireddi et al., 2019). In the absence of external stimuli, lack of TAK1 leads to the loss of cellular homeostasis, the release of inflammatory signals and PANoptosis (Malireddi et al., 2020a,b). A recent study linked TAK1 to aging; in that study, reduced TAK1 expression due to aging led to RIPK1-driven neurodegeneration that caused neuronal loss and behavioral defects (Xu et al., 2018).

## 9 Connections between PANoptosis, AD, and mitochondria

Current research on PANoptosis is mainly based on pathogen infection models; the occurrence of PANoptosis in association with non-infectious injuries, especially in the nervous system, remains unknown. A recent groundbreaking study conducted in Xiong's laboratory demonstrated the occurrence of PANoptosis-like cell death in retinal neurons during ischemia/reperfusion injury both *in vitro* and *in vivo* (Yan et al., 2023). Its specific manifestation is significant upregulation of the PANoptosome complex components caspase 1, caspase 8, and NLRP3. Although the assembly of the PANoptosome and the interactions among key molecules capable of regulating the three PCD pathways have not been systematically elucidated, the work of Xiong and his team strongly suggests that PANoptosis occurs as a form of neuronal death. However, neuronal death in AD is an extremely complex process, and different components of that process play different core roles within neurons and are capable of disrupting the fragile balance of the neuronal environment.

ZBP1 is upregulated in the brain tissue of AD rats and in AD neurons (Guo et al., 2023a). Silencing ZBP1 significantly reduces cell damage, oxidative stress, and inflammation in AD neurons and improves cognitive function in AD model rats (Guo et al., 2023a). ZBP1 can also be activated by mtDNA, and this activation induces the release of proinflammatory markers from microglia (Saada et al., 2022). Neuroinflammation is associated with neuronal necroptosis (Jayaraman et al., 2021). RIPK1 is a key mediator of neuronal death and inflammation and a critical target for treating AD (Yuan et al., 2019; Hugon and Paquet, 2021; Vissers et al., 2022). Phosphorylated MLKL is coexpressed with RIPK1 in CA1 pyramidal neurons of AD patients (Jayaraman et al., 2021). Neuron-specific RIPK1- or RIPK3-deficient mice exhibit significant protection against chronic traumatic brain injury and experience reduced neuronal apoptosis (Wehn et al., 2021). AIM2 can assemble into an inflammatory complex, leading to neuronal dysfunction and ultimately neurodegeneration (Hornung et al., 2009). AIM2 can also increase Aβ deposition, but it has little impact on memory function in 5xFAD mice (Wu et al., 2017). AβOs can activate AIM2 inflammasomes, leading to accumulation of the RIPK1 protein and disruption of mitochondrial autophagy (Cao et al., 2021). Furthermore, defective AIM2 inflammasome signaling leads to decreased neuronal death, but it responds to DNA damage-inducing agents and neuronal development processes (Lammert et al., 2020). Moreover, inhibition of TAK1 can reduce ischemia-induced neuronal apoptosis (Neubert et al., 2011; Wu et al., 2020).

Mitochondria are undoubtedly crucial for neuronal function and survival. Disruption of healthy neuronal mitochondrial function leads to an energy crisis, oxidative stress, and impaired cell signaling. The release of lethal mitochondrial contents such as ROS, mtDNA, and Cyto C is accompanied by the release of key upstream molecules that activate PANoptosis. Considering the close relationship between the neuronal survival environment and mitochondrial function and dynamics and the widespread reports of induction of neuronal death, including pyroptosis, apoptosis, and necroptosis, by imbalanced mitochondrial dynamics or mitochondrial dysfunction, it can be speculated that mitochondria may be a key factor in activating the unique PANoptosis pathway in neurons (Fricker et al., 2018). In the section that follows, we explore the potential mechanisms by which AβOs activate PANoptosis through mitochondria, focusing on imbalances in mitochondrial dynamics, disruptions in mitochondrial energy metabolism, and regulation of mitochondrial autophagy.

## 10 Mitochondria play a crucial role in initiating PANoptosis

### 10.1 Mitochondrial dynamics imbalance and reduced membrane integrity trigger PANoptosis

AβO has an affinity for membranes, forming ion channels on the membrane surface, resulting in a large influx of Ca^2+^ into mitochondria and leading to VDAC oligomerization, mPTP opening, and inner mitochondrial membrane permeability to solutes as large as 1.5 kDa (Norenberg and Rao, 2007). The opening of the mPTP and the release of mitochondrial contents, including intermembrane pro-apoptotic proteins such as Cyto C and apoptosis-inducing factor (AIF), as well as mtDNA, into the cytoplasm are lethal for neurons (Guan et al., 2021). By knocking out ANT and using cyclosporin A *in vivo*, CypD-dependent mPTP can be effectively inhibited, thereby suppressing apoptosis (Karch et al., 2019; Bround et al., 2023). In the presence of deoxyadenosine triphosphate, Cyto C can bind to apoptotic protease activating factor 1 (Apaf-1), leading to Apaf-1 polymerization and facilitating the formation of apoptosomes. Activated caspase 9 can then activate other caspases, such as caspase 3, thereby inducing cell apoptosis. Cyto C also triggers the inositol trisphosphate (IP3) receptor, thereby increasing cytoplasmic Ca^2+^ levels and ROS generation. Apaf-1 can also interact with caspase 4 to form a novel protein complex that further activates the caspase-3-GSDME signaling pathway, leading to rapid cell pyroptosis (Xu et al., 2021b). Activated caspases can also cleave GSDM proteins, releasing their N-terminal domains; the released domains bind to membrane lipids and continuously disrupt the inner and outer mitochondrial membranes while inducing cell pyroptosis (Liu et al., 2021; Miao et al., 2023). Unlike the apoptotic molecules Cyto C and AIF, mtDNA is a well-known DAMP that can activate inflammasomes, triggering cell pyroptosis (Marchi et al., 2023). mtDNA can enter the cytoplasm through the mPTP, which has a diameter of ~1.4 nm, and can be sensed by toll-like receptors, the NLRP3 inflammasome system, and the cyclic GMP-AMP synthase (cGAS)/stimulator of interferon gene (STING) signaling pathway, leading to the activation of downstream inflammatory factors (Decout et al., 2021). Although microglia are the main players in neuroinflammation caused by pyroptosis in the central nervous system, non-glial cells such as neurons can also independently participate in the inflammatory response through inflammasome signaling, and AβOs can even directly activate NLRP1 to induce neuronal pyroptosis (Tan et al., 2014; Yap et al., 2019). Tumor necrosis factor α (TNF-α) activated by the cGAS-STING signaling pathway binds to TNF receptors (TNFRs) on the cell surface, transmitting death signals through RIPK1 and RIPK3 and forming the RIPK1-RIPK3-MLKL necrosome. STING agonists can also activate STING-dependent PANoptosis (Messaoud-Nacer et al., 2022). Phosphorylation of RIPK3 and MLKL upregulates the expression of phosphoglycerate mutase 5 (PGAM5) on the mitochondrial membrane and increases CypD phosphorylation, resulting in cell necroptosis (Zhou et al., 2018). PGAM5 can also recruit Drp1 and activate its GTPase activity by dephosphorylating it at the Ser637 site (Wang et al., 2012). Drp1 activation leads to mitochondrial fragmentation, an early and obligatory step in necrosis.

### 10.2 Damage to mitochondrial aerobic respiration triggers PANoptosis

Hexokinase 1 (HK-1) catalyzes the phosphorylation of glucose at the hydroxyl group at the sixth position. This reaction is the first step of glycolysis. Excess phosphorylation of Drp1 by AβOs can induce glycolysis defects in AD models by inhibiting HK-1, leading to downstream NLRP3-related molecular signaling and the induction of cell apoptosis (Zhang et al., 2020). HK-2 can dissociate from VDAC and promote VDAC oligomerization, resulting in assembly of the NLRP3 inflammasome and triggering cell apoptosis (Baik et al., 2023). AβOs can also cause reduced enzyme activity in the TCA cycle, particularly reducing the activity of succinate dehydrogenase (SDH; also known as complex II; Swerdlow, 2012). SDH is linked to two important physiological metabolic processes: the TCA cycle and OXPHOS (Du et al., 2023). Decreased activity of SDH leads to the accumulation of succinate, which is rapidly oxidized, driving ROS generation through reverse electron transfer to complex I, resulting in PANoptosis in cells (Chouchani et al., 2014; Shi et al., 2023b). This phenomenon is reversed by reverse electron transfer inhibitors (Shi et al., 2023b).

In eukaryotic cells, aerobic respiration relies on the OXPHOS system located on the mitochondrial cristae. This system includes the electron transport chain composed of transmembrane protein complexes I-IV. AβOs disrupt the structure of mitochondrial cristae, leading to the accumulation of ROS. Since mtDNA is located close to the ETC complexes on the inner membrane and lacks histone protection, mtDNA is the primary target of ROS attack, resulting in oxidative damage to mtDNA. In AD, oxidative damage to mtDNA directly inhibits the synthesis of mitochondrial DNA-encoded proteins, affecting their proper expression, reducing the number of mitochondria, disrupting intracellular energy metabolism, and causing abnormal apoptosis of neurons. Additionally, oxidative damage-mediated 8-hydroxyguanine and formamidopyrimidine modifications lead to G:C → T:A transversion mutations in mtDNA, resulting in the formation of a mutated form of mtDNA that assumes a Z-DNA conformation (Yasui et al., 2014). The Z-DNA structure is characterized by a left-handed helix and a highly twisted structure, causing it to present a zigzag appearance (Herbert, 2019). ZBP1, a PANoptosome complex, can specifically recognize and bind to Z-DNA, thereby activating cell-programmed necrosis and pyroptosis pathways dependent on the RIPK3-MLKL and ZBP1-NLRP3 pathways (Ravichandran et al., 2019; Jiao et al., 2020; Zheng and Kanneganti, 2020; Zhang et al., 2022; Nassour et al., 2023). A recent study indicated that instability of mtDNA drives ZBP1-cGAS-RIPK-dependent IFN-I signaling *in vitro*, suggesting that the ZBP1-cGAS complex may act as a specialized pattern recognition receptor for Z-type mtDNA (Lei et al., 2023). ZBP1 can also activate inflammatory signaling pathways that are independent of cell death mediated by non-degradative ubiquitin chains, which requires RIPK1 and RIPK3 as molecular scaffolds (Peng et al., 2022). Oxidatively damaged mtDNA is either repaired by 8-oxoguanine DNA glycosylase or cleaved into 500–650 bp fragments by an endonuclease; it then exits mitochondria through channels dependent on mPTP and VDAC and activates the NLRP3 inflammasome. NLRP3 can sense low-methylated CpG motifs in mtDNA and participate in the activation of caspase 1, promoting the activation of IL-1 and IL-18(Zhong et al., 2018).

ROS can also regulate signal transduction related to necroptosis and cell death by acting as an upstream signal that activates the PANoptosome (Vandenabeele et al., 2010; Shindo et al., 2013; Fulda, 2016). Three cysteine residues (C257, C268, and C586) in RIPK1 can sense ROS, thereby activating RIPK1 S161 phosphorylation, and RIPK1 is most likely the only target of ROS in necrotic apoptosis (Zhang et al., 2017). This phosphorylation process allows RIPK3 to effectively recruit RIPK1 to form functional necrosomes, thereby activating necroptosis. ROS can also mediate the transition from apoptosis to necroptosis in a high-glucose environment (Deragon et al., 2020). It is known that AβOs disrupt neuronal energy metabolism homeostasis and compete with neuronal IR for binding; this leads to reduced neuronal glucose uptake and impairment of aerobic glycolysis and the TCA cycle and results in symptoms of insulin resistance and a low rate of glucose utilization in the brains of AD patients (De Leon et al., 1983; Gali et al., 2019). High levels of ROS oxidize RIPK1, causing it to form high-molecular-weight oligomers and promoting the formation of necrosomes and the activation of RIPK1, RIPK3, and MLKL (Deragon et al., 2020). In addition, ROS-mediated formation of disulfide bonds between MLKL proteins contributes to MLKL oligomerization, thereby promoting necrotic apoptosis (Liu et al., 2017). RIPK3, in turn, can directly phosphorylate the E3 subunit of the pyruvate dehydrogenase complex, further enhancing mitochondrial aerobic respiration and the production and accumulation of ROS (Yang et al., 2018). Elevated levels of ROS, particularly oxidized cardiolipins (CLs, lipids that are present only in the IMM and are susceptible to oxidative damage), also induce cellular oxidative stress and disrupt cell structure and mitochondrial outer membrane permeability, leading to lipid peroxidation and disruption of mitochondrial membrane structure and function. Moreover, ROS can cause nuclear DNA damage and induce excessive activation of PARP-1, resulting in extensive breakdown of NAD to nicotinamide and poly (ADP-ribose; PAR). On the one hand, depletion of the mitochondrial NAD pool can lead to disruption of mitochondrial energy metabolism, exacerbating ROS and apoptosis signal generation. On the other hand, PAR induces AIF nuclear translocation, cleaves chromosomal DNA, and initiates the parthanatos cell death pathway. Notably, parthanatos does not cause cell membrane or organelle swelling, cell lysis, or RIPK1 activation; thus, this process needs to be distinguished from classical necroptosis (Huang et al., 2022).

### 10.3 Mitochondrial autophagy damage triggers PANoptosis

Neurons are a class of highly energy-demanding cells. To meet cellular energy demands, the renewal and maintenance of a healthy and active mitochondrial pool is essential (Cai and Tammineni, 2016). Mitochondrial autophagy, known as mitophagy, is a highly conserved cellular process in eukaryotic cells. An irregular distribution of mitochondria, morphological damage (fragmentation, ultrastructural damage), and alterations in bioenergetics (membrane potential, cytochrome c oxidase activity, decreased ATP production) lead to the accumulation of unhealthy mitochondria, inducing mitophagy to selectively target damaged mitochondria (Onishi et al., 2021). Consequently, the efficient removal of damaged mitochondria through mitophagy is crucial for maintaining bioenergetic homeostasis in neurons. Typically, mitophagy does not directly participate in cellular apoptosis, pyroptosis, or necroptosis. Unlike other processes involved in maintaining mitochondrial homeostasis, mitophagy is more involved in mitochondrial quality control, clearing mitochondrial waste and unhealthy mitochondria and thereby indirectly regulating cell fate. Mitochondria with excessively damaged mtDNA that cannot be repaired through fission/fusion, as well as depolarized mitochondria and those with damaged ETC, are preferentially cleared by mitophagy. Mitophagy is primarily mediated by PTEN-induced kinase 1 (PINK1)-Parkin and by the assembly of ubiquitin chains on the mitochondrial outer membrane (Wang et al., 2023). PINK1 acts as a sensor of mitochondrial damage, Parkin acts as a signal amplifier, and ubiquitin chains act as signal effectors. PINK1 can sense depolarized mitochondria. The expression of mitophagy-related proteins such as BCL2-like 13 (Bcl2L13), PINK1, BCL2/adenovirus E1B 19 kDa protein-interacting protein 3-like (BNIP3L), phosphorylated UNC-51-like kinase 1 (p-ULK1), phosphorylated TANK-binding kinase 1 (p-TBK1), and FUN14 domain-containing protein 1 (FUNDC1) is downregulated in the brains of AD patients and their derived induced pluripotent stem cells, indicating defects in the mitophagy pathway in AD (Fang et al., 2019). The downregulation of autophagy capacity in AD may be related to AβOs. Stimulation with AβOs leads to excessive mitochondrial fission, increasing the number of depolarized mitochondria. Depolarized mitochondria recruit high levels of Parkin (Ye et al., 2015; Wang et al., 2020). This is a necessary step in separating damaged mitochondria before they are engulfed by autophagosomes. AβOs lead to mPTP opening and occupy the TOM complex, allowing them to enter mitochondria. This process inhibits the import of PINK1 into mitochondria, preventing PINK1 from trans-autophosphorylating through TOM, and PINK1 is thus unable to execute subsequent mitophagy programs (Pinho et al., 2014; Gan et al., 2022). Mitochondrial-derived vesicles (MDVs) carrying damaged mitochondrial components fuse with lysosomes, completing the hydrolytic degradation of MDVs and preventing oxidatively damaged mtDNA from triggering apoptosis and pyroptosis signaling pathways (Mclelland et al., 2014; Sugiura et al., 2014). The energy stress caused by AβO interference also restricts mitochondrial autophagy. AMP-activated protein kinase (AMPK) is the main factor that regulates PINK1-Parkin-mediated mitophagy (Herzig and Shaw, 2018). When ATP levels decrease during energy stress, AMPK is activated and catalyzes the phosphorylation of ULK1 and ULK2 (two homologs of Atg1), thereby activating mitophagy (Laker et al., 2017). AβOs induce mitochondrial damage and may further disrupt DNA through energy depletion and the release of ROS (Cardinale et al., 2012; Shadfar et al., 2022). Typically, under conditions of energy stress, AMPK directly phosphorylates key factors, such as peroxisome proliferator-activated receptor gamma coactivator 1-alpha (PGC-1α) and mitochondrial transcription factor A (TFAM), in multiple pathways to maintain mitochondrial biogenesis and restore energy balance (Abu Shelbayeh et al., 2023). However, when DNA damage occurs, the phosphorylation level of AMPK is reduced, slowing mitochondrial biogenesis and autophagy regulation, and leading to a decrease in mitochondrial quality and quantity (Guo et al., 2023b). Therefore, it can be inferred that mitochondrial biogenesis and mitophagy collectively help maintain mitochondrial homeostasis, aiding neurons in coping with attack by AβOs. The AMP/ATP ratio, the Ca^2+^ level, and the NAD^+^/NADH ratio are the factors that are most relevant to the regulation of mitochondrial autophagy and biogenesis. These ratios undergo abnormal changes during AD, and they are typical manifestations of mitochondrial damage caused by AβOs. Additionally, silent information regulator 1 (Sirt1) deacetylates and activates PGC-1α in response to depletion of the mitochondrial NAD^+^ pool, reducing mitochondrial biogenesis and autophagy (Biel et al., 2016). However, when DNA damage occurs, Sirt1 competes with poly (ADP-ribose) polymerase (PARP) for NAD^+^, thereby inducing apoptosis.

CL can mediate non-ubiquitin-dependent mitochondrial autophagy. CL binds to the hexamer membrane-integrated protein NDPK-D and then externalizes from the IMM to the OMM, increasing the amount of CL on the OMM; subsequently, CL is recognized by microtubule-associated protein 1A/1B-light chain 3A (LC3A) and LC3B, which serve as “eat me” signals for mitochondrial autophagy in neuronal cells (Chu et al., 2013; Kagan et al., 2016). NLRP3 binds directly to CL lipids and plays a crucial role in the activation of the NLRP3 inflammasome (Iyer et al., 2013). Due to the lipophilicity of AβOs, CL, which contains a glycerol backbone that is connected to phospholipids and which stabilizes respiratory chain proteins in the IMM, may be permeabilized by AβOs (Camilleri et al., 2013; De La Ballina et al., 2020). Specific CL binding sites have been detected in complexes I, III, and IV, and these sites are crucial for the stability and enzymatic activity of these complexes (Lange et al., 2001; Musatov and Sedlák, 2017). CL-dependent mitochondrial autophagy promotes the selective degradation of SDHB, SDHC, and SDHD, thereby contributing to metabolic reprogramming during inflammation (Reynolds et al., 2023). Excessive ROS can also oxidize CL, further affecting the activity of complexes I, III, and IV, exacerbating oxidative stress under AβO pressure and activating apoptosis, pyroptosis, and necroptosis signaling pathways (Paradies et al., 2000, 2001, 2002).

In summary, the toxic effects of AβOs on mitochondria weaken neuronal compensatory mechanisms. Failure to degrade damaged mitochondria in a timely manner triggers a vicious cycle of sustained PANoptosis activation.

## 11 Discussion and additional considerations

In 1984, Glenner and Wong extracted Aβ from pathological plaques in the brains of AD patients (Glenner and Wong, 1984). In 1991, Goate et al. (1991) detected mutations in the APP gene in families with hereditary early-onset AD. In 1992, Hardy and Higgins proposed the amyloid hypothesis (Hardy and Higgins, 1992). In 1998, Lambert et al. first reported the spontaneous formation of soluble Aβ oligomers (Lambert et al., 1998). Research on the toxicity of Aβ oligomers has since become an important research direction in the field of AD. However, much remains to be understood about the pathogenesis of AD and the discovery of therapies for treatment of this disease.

PANoptosis is a key feature of apoptosis, pyroptosis, and necroptosis and will undoubtedly be an important focus of future research on programmed cell death. However, key evidence for the occurrence of PANoptosis in neurons under AβO pressure is still lacking. Based on discoveries made in clinical, epidemiological, genetic, and molecular biological research, it appears that mitochondria, important organelles that regulate cell “life” and “death,” play a crucial role in the occurrence and development of AD, and this idea may constitute a breakthrough in understanding neuronal PANoptosis. Under conditions involving various physiological or pathological stimuli, imbalanced mitochondrial dynamics lead to mitochondrial fragmentation, damage to mitochondrial structures such as cristae, decreased ATP production capacity, increased membrane permeability, and decreased membrane potential. This can lead to the release of mtDNA, ROS, and Cyto C during the process of apoptosis. These materials act as upstream signaling molecules to initiate PANoptosis and participate widely in physiological processes such as neuronal apoptosis, aging, and autophagy. However, several unresolved questions remain to be answered.

First, considering the complex crosstalk that exists among PCD pathways, simply inhibiting the signaling that brings about apoptosis, pyroptosis, and necroptosis may not allow researchers to adequately characterize PANoptosis. Due to the non-redundancy of PANoptosome function, targeting any aspect of PCD may lead to the enhancement of certain molecular components by other components or may even be fatal (Dillon et al., 2014; Sundaram et al., 2022). Thus, future research should be inclusive and should consider the overall biological effects of PANoptosis to allow investigators to mechanistically understand its regulation. For example, multiomics analysis and machine learning can reveal connections and correlations among different biological components and are adept at identifying complex patterns in large datasets. These patterns may not be apparent when each component is studied individually but can lead to the discovery of new relationships and pathways within biological systems. This is particularly applicable to PANoptosis research and the discovery of novel PANoptosomes. Furthermore, recent studies have also demonstrated the usefulness of whole-genome CRISPR screening and expansion microscopy methods in screening for potential PANoptosomes and evaluation of the production and assembly of PANoptosomes under PANoptosis induction (Wang et al., 2022; Malireddi et al., 2023).

Second, past developments in Aβ-based therapies have focused mainly on plaque clearance and have overlooked AβOs. However, even some AβO-selective high-affinity drugs such as the FDA-approved drugs aducanumab and donanemab, which activate the immune system in patients and cause it to clear AβOs and amyloid plaques from the brain, require high doses to neutralize a small fraction of AβOs (Sevigny et al., 2016; Knopman et al., 2021). Additionally, some natural plant sugars and synthetic drugs that inhibit AβO formation, such as scyllo-inositol and tramiprosate, lack significant efficacy in phase III clinical trials (Santa-Maria et al., 2007; Salloway et al., 2011). The greatest challenge in this area lies in the fact that while AβOs are neurotoxic, soluble Aβ at physiological levels also has inherent biological functions. Research on neuronal PANoptosis will aid in the identification of treatment targets and the development of new drugs. Imbalances in mitochondrial dynamics or a breakdown in quality control under conditions of AβO interference may release important components that govern PANoptosis, and the factors that coregulate these three cell death pathways may be viable biological targets for therapy. Therefore, considering the important role of mitochondria at all stages of AD, even the pre-MCI stage, mitochondrial dysfunction may play a crucial role as a primary or secondary event in AD, and targeting mitochondrial function may represent promising therapeutic targets. Emerging areas have focused on the targeting of multiple mitochondrial functions by mitochondrial nutrients, as well as on stem cell therapy, to maintain mitochondrial homeostasis while minimizing side effects. However, oligomers are inherently highly dynamic and difficult to separate, making the research challenging (Benilova et al., 2012). Attention should be given to the aggregation states Aβ is in before and after it enters mitochondria. The source of AβOs also needs to be considered. Synthetic AβOs have the advantages of high compound purity and the absence of contaminants, whereas AβOs extracted from organisms have more impurities; this can affect AβO aggregation but may allow better simulation of biological processes (Teplow, 2013). Therefore, caution should be exercised in selecting the AβOs used in experiments.

Third, the discovery of PANoptosis greatly broadens our understanding of PCD. Although PANoptosis was first discovered in a case of IAV infection, restricting the study of PANoptosis to infection-related research is unwise. The non-redundancy of the PANoptosome emphasizes that it is unlikely to cause only one type of cell death, particularly in the highly developed brain with its mitochondria. For example, iron death and copper death are caused by mitochondrial dysfunction, and non-caspase-dependent parthanatos and the recently discovered disulfide death pathway also occur in cells. PANoptosis may be only the tip of the iceberg among many forms of cell death. The coordination among various death pathways suggests that we should pay attention to the regulation of other forms of cell death while studying PANoptosis.

## 12 Conclusion

In conclusion, the disruption of mitochondrial dynamics and mitochondrial function by AβOs may trigger neuronal PANoptosis during the occurrence of AD. Continued attention to the latest evidence of PANoptosis in neurons and AD-related research will provide new perspectives for the research and treatment of neurodegenerative diseases such as AD.

## Author contributions

XM: Conceptualization, Visualization, Writing—original draft, Writing—review & editing. QS: Visualization, Writing—review & editing. ZL: Writing—review & editing. XL: Writing—review & editing. YW: Writing—review & editing. JL: Project administration, Supervision, Writing—review & editing.

## Glossary

α7nAChR, alpha 7-nicotinic acetylcholine receptor; αKG, α-ketoglutarate; αKGDH, α-ketoglutarate dehydrogenase; AMY3, Amylin 3 receptor; Aβ, β-amyloid protein; AβO, Aβ oligomer; ABAD, Aβ-binding alcohol dehydrogenase; AD, Alzheimer's disease; ADP, Adenosine diphosphate; AIF, apoptosis-inducing factor; AIM2, absent in melanoma 2; AMPA,α-amino-3-hydroxy-5-methylisoxazole-4-propionic acid receptor; AMPK, AMP-activated protein kinase; ANT, adenine nucleotide translocator; Apaf-1, apoptotic protease activating factor 1; APP, amyloid precursor protein; ASC, apoptosis-associated speck-like protein; ATP, adenosine triphosphate; BACE1, β-site amyloid precursor protein cleaving enzyme-1; Bcl2L13, BCL2 like 13 Gene; BNIP3L, BCL2/Adenovirus E1B 19 kDa protein-interacting protein 3-like; cGAS, Cyclic GMP-AMP Synthase; CL, cardiolipins; CoA, acetyl coenzyme A; Cyp D, cyclophilin D; Cyto C, cytochrome C; DAMP, danger-associated molecular patterns; EphA4, Ephrin type A receptor 4; EphB2, Ephrin type B receptor 2; ETC, electron transport chain; FcγRIIb, Immunoglobulin gamma Fc region receptor II-b; Fis1, fission protein 1; FUNDC1, FUN14 domain-containing protein 1; FZD, Frizzled receptors; GLUT3, glucose transporter 3; Gpc4, Glypican 4; Grp75, glucose-regulated protein 75; GSDMD, gasdermin D; GTPase, guanosine triphosphatase; HK-1, hexokinase 1; IAV, influenza A virus; IL, interleukin; IMM, inner mitochondrial membrane; IMS, intermembrane space; IP3, inositol trisphosphate; IR, insulin receptor; LC3A, microtubule-associated protein 1A/1B -light chain 3A; LILRB2, leukocyte immunoglobulin-like receptor subfamily B member 2; MAM, mitochondria-associated endoplasmic reticulum membranes; MCI, mild cognitive impairment; MDV, mitochondrial-derived vesicles; Mff, mitochondrial fission factor; Mfn1, mitochondrial fusion protein 1; Mfn2, mitochondrial fusion protein 2; mGluR5, metabotropic glutamate receptor 5; MiD 49, mitochondrial dynamics proteins of 49 kDa; MLKL, mixed lineage kinase domain-like protein; MMSE, mini-mental state examination; mPTP, mitochondrial permeability transition pore; mtDNA, mitochondrial DNA; NAD, nicotinamide adenine dinucleotide; NADPH, nicotinamide adenine dinucleotide phosphate; NgR, Nogo receptor; NKAα3, neuron-specific NaK ATPase α3 subunit; NLs, Neuroligins; NLRP3, NOD-like receptor thermal protein domain associated protein 3; NMDAR, N-methyl-D-aspartic acid receptor; OMM, outer mitochondrial membrane; OPA1, optic atrophy 1; OSCP, oligomycin sensitivity conferring protein; OXPHOS, oxidative phosphorylation; p75NTR, p75 neurotrophin receptor; PAMP, pathogen-associated molecular patterns; PAR, Poly ADP-Ribose; PARP, Poly ADP-ribose polymerase; PCD, programmed cell death; PDH, pyruvate dehydrogenase; PGAM5, phosphoglycerate mutase 5; PGC-1α, peroxisome proliferator-activated receptor gamma coactivator 1-alpha; PGRMC1, progesterone receptor membrane component 1; PINK1, PTEN-induced kinase; PirB, paired Ig-like receptor B; PITRM1, pitrilysin metallopeptidase 1; PreP, presequence protease; PrPc, cellular prion protein; PS1, presenilin-1; PS2, presenilin-2; p-TBK1, phosphorylated TANK-binding kinase 1; p-ULK1, phosphorylated UNC-51-like kinase 1; RAGE, receptor of advanced glycation endproducts; RHIM, RIP homotypic interaction motif; RIPK1, receptor-interacting protein kinase 1; ROS, reactive oxygen species; sAPPα, soluble APP fragment α; SDH, succinate dehydrogenase; Sirt1, Silent information regulator 1; STING, stimulator of interferon gene; TAK1, transforming growth factor-β activated kinase 1; TCA, tricarboxylic acid; TFAM, transcription factor A; TIM, translocase of the inner membrane; TNF-α, tumor necrosis factor α; TNFRs, TNF receptors; TOM, translocase of the outer membrane; TPK1, tau protein kinase 1; TRPM2, transient receptor potential melastatin family subtype 2; VDAC, voltage-dependent anion channel; ZBP1, Z-DNA-binding protein 1.
